# Piezo1-Fstl1 Axis in Fracture Healing: Modulation of the Chondrocyte Inflammation-ROS-Mitochondrial Damage Cascade and Application of Smart Delivery System

**DOI:** 10.7150/ijbs.125814

**Published:** 2026-02-18

**Authors:** Tao Zhang, Haoran Wang, Guangzhao Hou, Siming Jia, Kai Ding, Qian Xiao, Jinbo Liu, Yajiang Dai, Gang Lv, Zhiyong Hou, Yingze Zhang, Juan Wang, Hongzhi Lv, Wei Chen

**Affiliations:** 1 Department of Orthopaedic Surgery, Hebei Medical University Third Hospital; Shijiazhuang, Hebei 050051, China.; 2 Key Laboratory of Biomechanics of Hebei Province; Shijiazhuang, Hebei 050051, China.; 3 NHC Key Laboratory of Intelligent Orthopaedic Equipment; Shijiazhuang, Hebei 050051, China.; 4 Engineering Research Center of Orthopedic MinimallyInvasive Intelligent Equipment, Ministry of Education; Shijiazhuang, Hebei 050051, China.; 5 The Fourth Clinical Medical College of Xinjiang Medical University, Traditional Chinese Medicial Hospital of Xinjiang Uygur Autonomous Region; Urumqi, Xinjiang 830001, China.

**Keywords:** fracture healing, Piezo1-Fstl1, intelligent delivery system, inflammatory reaction, mitochondrial oxidative stress

## Abstract

This study investigated the regulatory role of an intelligent drug delivery system in promoting fracture healing via Piezo1-Fstl1 signaling axis. It also verified its modulation of chondrocyte inflammatory response, mitochondrial oxidative stress, and osteoblast differentiation. Inflammation triggers the accumulation of pro-inflammatory factors, and reactive oxygen species (ROS) in chondrocytes. This leads to oxidative damage in mitochondria, a decrease in mitochondrial membrane potential (MMP), and the induction of mitochondrial permeability transition pore (mPTP) opening, thereby hindering fracture healing. Single-cell RNA sequencing revealed that Piezo1 deficiency markedly upregulated the expression of follistatin-like protein 1 (Fstl1) in chondrocytes. This upregulation exacerbated chondrocyte inflammation and impaired the chondrocyte-to-osteoblast differentiation. Inhibition of Fstl1 attenuated the inflammatory response and ROS accumulation associated with Piezo1 deficiency, alleviated mitochondrial oxidative stress, and improved mitochondrial function and homeostasis. It also restored mitochondrial cristae ultrastructure, thereby improving MMP and mitochondrial activity. This intervention concurrently upregulated osteogenic markers and accelerated endochondral ossification. Based on these, we developed a HA-PBA/TA self-healing hydrogel incorporating chondrocyte-targeting lipid nanoparticles (C-LNP^@Fstl1^) to suppress Fstl1 expression. Local injection of this hydrogel into murine femoral fracture sites significantly reduced inflammatory cytokines in callus tissue and promoted fracture healing, offering new insights and therapeutic strategies for fracture treatment.

## Introduction

As traumatic fracture cases continue to rise worldwide, the incidence of delayed union and nonunion following surgery has similarly increased^1^. Patients with fracture nonunion often require revision surgical procedures, which significantly hinder functional recovery, reduce quality of life, and escalate medical costs; these factors further exacerbate the financial burden on families^2^. Consequently, elucidating the molecular mechanisms underlying fracture healing and developing targeted interventions using smart, responsive nanomaterials—advanced materials designed to interact dynamically with biological systems—represent critical clinical challenges and key research priorities^3^.

Endochondral ossification serves as the primary mechanism for long bone fracture healing, critically important in unstable, defect-associated, or non-anatomically reduced fractures^4^. Our prior lineage tracing experiments confirmed that chondrocytes undergo transdifferentiation into osteoblasts during fracture healing^5^. Piezo1 activation promotes this chondrocyte-to-osteoblast transdifferentiation, thereby accelerating fracture repair^6^. Conversely, Piezo1 deficiency in chondrocytes induces marked mitochondrial dysfunction^7^. Maintenance of functional mitochondria is essential for endochondral ossification during fracture healing; impaired mitochondrial activity disrupts oxidative phosphorylation, severely compromising adenosine triphosphate (ATP) production and obstructing chondrocyte transdifferentiation^8^, ^9^. In addition, inflammatory responses also hinder endochondral ossification^10^. Inflammatory responses disrupt chondrocyte microenvironmental homeostasis, elevating reactive oxygen species (ROS) levels^11^. This induces mitochondrial oxidative stress, triggering mitochondrial permeability transition pore (mPTP) opening and impairing mitochondrial activity, then blocking chondrocyte transdifferentiation into osteoblasts and contributing to delayed union or nonunion of fractures^12^. Our single-cell RNA sequencing (scRNA-seq) analysis of day-14 fracture callus revealed pronounced upregulation of follistain like protein 1 (Fstl1) in chondrocyte-specific Piezo1 knockout (Piezo1*^Col2a1^*) mice. Fstl1, functions as a novel inflammatory protein that activates the NF-κB signaling pathway to exacerbate chondrocyte inflammation and promote extracellular matrix degradation^13^, ^14^. However, no studies exist on the interaction between Piezo1 and Fstl1 in regulating endochondral ossification during fracture healing. Therefore, this study aims to investigate the crosstalk among Piezo1-Fstl1 signaling axis, chondrocyte inflammation, ROS expression, mitochondrial function, and osteogenic differentiation, elucidating its regulatory role in endochondral ossification during fracture healing.

Lipid nanoparticle hydrogels (LNPHs) represent an advanced drug delivery system that combines lipid nanoparticles (LNPs) with hydrogel matrix^15^, ^16^. This system overcomes key drawbacks of traditional carriers by integrating the high drug-loading efficiency of liposomes with sustained-release hydrogel properties^17^, ^18^. Compared to conventional delivery platforms, LNPHs exhibit superior sustained drug release, targeted delivery capability, bioactive cargo protection, and enhanced biocompatibility, making them highly promising for transformative applications in precision medicine and regenerative medicine^19^, ^20^. In situ injection of LNPHs loaded with targeted therapeutics into fracture sites significantly enhances treatment efficacy^21^.

In the current study, we first confirmed Piezo1 deficiency induced chondrocytic inflammatory factors, compromised endochondral ossification progression, and identified Fstl1 as a key downstream effector through scRNA-seq. Next, we demonstrated that inducing chondrocyte inflammation promotes ROS expression, exacerbating mitochondrial oxidative stress, inducing mPTP opening, impairing mitochondrial function, and blocking chondrocyte transdifferentiation into osteoblasts. And suppressing Fstl1 in chondrocytes significantly reduces inflammatory factors and ROS generation, ameliorates mitochondrial oxidative stress and dysfunction, and promotes endochondral ossification. Finally, we developed a drug delivery system of Hyaluronic acid-phenylboric acid/Tannic acid (HA-PBA/TA) self-healing hydrogel loaded with chondrocyte-targeting lipid nanoparticles (C-LNP^@Fstl1^) to suppress Fstl1 expression. In situ injection of this system into murine femoral fracture sites for targeted therapy markedly accelerated fracture healing. Collectively, our findings point to a new perspective on the role of chondrocytes in endochondral ossification.

## Materials and Methods

### Experimental animals

All *in vivo* experiments were conducted on 12-week-old male mice. Wild-type C57BL/6J mice were obtained from the SiPeiFu Biotechnology Co. Ltd. (Beijing, China). *Col2a1*-CreERT2 mice were obtained from Cyagen Biosciences Inc. (Guangzhou, China). The Piezo1*^f/f^* mice were provided by Professor Weiguo Zou (Hainan Academy of Medical Sciences and School of Basic Medicine, Hainan Medical University). To generate Piezo1*^Col2a1^* mice, Piezo1*^f/f^* mice were mated with *Col2a1*-CreERT2 mice, and filial-generation 1 (F1) littermates were mated with each other. The genotypes of Piezo1*^Col2a1^* mice were determined by genotyping all filial-generation 2 (F2) offspring mice. The primers used for each genotype are listed in Table S1. All animal experiments were approved by the Ethics Committee of the Hebei Medical University Third Hospital (Z2024-005-2).

### Femoral fracture model

The surgery was performed as previously described^22^. Briefly, mice were anesthetized with isoflurane gas (3% for induction and 1% for maintenance). Next, the right femur was sterilized and then a transverse fracture was created at the mid-shaft, and the fracture was fixed by inserting a sterilized 23-gauge needle into the medullary cavity. For the gene mice, tamoxifen (75 mg/kg) was dissolved in corn oil and administered subcutaneously for 5 consecutive days to induce the deletion of Piezo1 in chondrocytes. The first injection was performed on the 5^th^ day after surgery, as chondrocytes in the callus first appeared on postoperative day 5^22^. Complete femoral samples were harvested at 14^th^ day.

### ScRNA-seq

For the scRNA-seq experiments in this study, we used callus tissues from Piezo1*^Col2a1^* (n = 4) and Piezo1*^f/f^* (n = 6) mice 14 days post-surgery. Follow the description in the previously published article. All scRNA-seq data were uploaded to the National Center for Biotechnology Information (NCBI) Gene Expression Omnibus database (accession number: GSE266774).

### Micro-CT analysis

The removed fresh femoral specimens were scanned using a SkyScan 1176 micro-CT instrument (Micro-CT; 50 kV, 500 μA, and 9 μm/pixel). Micro-CT scan images were 3D reconstructed using the NRecon software (version 1.6, SkyScan; Microphotonics Inc., Allentown, PA, USA). Quantitative analysis of the fracture callus tissue images was performed using CTAn software (version 1.9, SkyScan). The parameters for micro-CT analysis were bone volume (BV), total volume (TV), bone volume fraction (BV/TV), trabecular number (Tb. N), trabecular thickness (Tb. Th.), and trabecular separation (Tb.Sp).

### Staining of tissue sections

Femoral samples were decalcified for 21 days using an EDTA decalcifying solution (EDTA, Solarbio Science & Technology, Beijing, China). After decalcification, samples were dehydrated and embedded in paraffin. A series of 5 μm consecutive paraffin-embedded sections were cut from each sample for hematoxylin-eosin (HE), Safranin O and Fast Green (SO/FG), and Immunohistochemistr (IHC) staining. The HE and SO/FG staining were performed using commercial kit (G1120, Solarbio, Beijing, China; G1371, Solarbio, Beijing, China) following standardized histopathological protocols. For IHC staining, the operation was performed as previously described. Briefly, after antigen retrieval, the specimens were blocked using goat serum. Diluted primary antibody was added and incubated at 4 °C overnight. Then enzyme-conjugated goat anti-rabbit/mouse IgG polymer added dropwise. Appropriate amount of freshly prepared DAB solution was added, and the samples were counterstained with haematoxylin for 1 min until the nuclei appeared blue, and the staining was immediately stopped. The primary antibodies used were Piezo1 (1:100, 15939-1-AP, Proteintech, USA), Fstl1 (1:100, ab223287, Abcam, UK), Collagen Type I (1:100, 14695-1-AP, Proteintech, USA), Osteopontin (1:200, 22952-1-AP, Proteintech, USA), RUNX2 (1:200, sc-390351, Santa Cruz Biotechnology, USA), Osteocalcin (1:200, DF12303, Affinity Biosciences, USA), NF-κB p65 (1:200, 10745-1-AP, Proteintech, USA), TNF-α (1:200, 17590-1-AP, Proteintech, USA), and IL-1β (1:200, 26048-1-AP, Proteintech, USA).

### Cell culture and osteogenic differentiation

Piezo1*^WT^* and Piezo1^-/-^ ATDC5 cells were obtained from Cyagen Biosciences. Osteogenic differentiation of Piezo1*^WT^* and Piezo1^-/-^ ATDC5 cells was performed following the protocol reported by Hendrickx *et al.*^23^. After 7 days of osteogenic induction, ALP staining was performed using a BCIP/NBT alkaline phosphatase chromogen kit (Beyotime). After 21 days of osteogenic induction, calcium deposition was visualised using Alizarin Red staining (Solarbio Science & Technology). RNA and protein samples were extracted from the cells and collected after 14 days of osteogenic induction.

### Cell transfection

We used the shFstl1 plasmid for cell transfection. shFstl1 was designed and constructed by Shanghai Jikai Gene Technology Co., Ltd. Three shFstl1 interference sequences were designed (Table S2) and cloned into a hU6-MCS-CMV-Puromycin vector to construct the plasmid. Follow the description in the previously published article. Cell plasmid DNA transfection was performed using Lipo6000™ transfection reagent (C0526, Beyotime, China). Through WB analysis, the shFstl1 plasmid with the most effective interference was selected for subsequent cell experiments.

### qPCR experiments

Total RNA was extracted from cells using the RNeasy RNA extraction kit (Thermo Fisher Scientific, CA, USA). Reverse transcription was performed using GoScript™ Reverse Transcription Mix and Oligo dT primers (Promega Corporation, Madison, WI, USA). The mRNA expression levels were analysed by RT-PCR using a One Step RT-qPCR Kit (Sangon Biotech, Shanghai, China). Primers used for qPCR are listed in Table S3. The 2^-ΔΔCt^ method was used for data analysis.

### WB experiments

Details of the method for western blot analysis have been previously described^22^. The primary antibodies used were Piezo1 (1:1000, 15939-1-AP, Proteintech, USA), Fstl1 (1:1000, ab223287, Abcam, UK), Collagen Type I (1:1000, 14695-1-AP, Proteintech, USA), Osteopontin (1:1000, 22952-1-AP, Proteintech, USA), RUNX2 (1:1000, sc-390351, Santa Cruz Biotechnology, USA), Osteocalcin (1:1000, DF12303, Affinity Biosciences, USA), NF-κB p65 (1:1000, 10745-1-AP, Proteintech, USA), TNF-α (1:1000, 17590-1-AP, Proteintech, USA), MFN1 (1:1000, 13798-1-AP, Proteintech, USA), MFN2 (1:1000, 12186-1-AP, Proteintech, USA), OPA1 (1:1000, 27733-1-AP, Proteintech, USA), DRP1 (1:1000, 12957-1-AP Proteintech, USA), β-Tubulin (1:1000, ab7291, Abcam, USA), β-actin (1:1000, 20536-1-AP, Proteintech, USA), and GAPDH (1:1000, 10494-1-AP, Proteintech, USA).

### Cell viability assay

We employed the Super-Enhanced Cell Counting Kit-8 (C0048S, Beyotime, China) for the assay. Briefly, cells (5 × 10^3^ cells/well) were resuspended and seeded into 96-well plates. Add 100 μL of fresh medium containing different LPS concentrations (0, 5, 10, 20, 40, 80, 100, 150,200 ng/mL) to each well, followed by 24-hour incubation at 37 °C. Subsequently, add 10 μL of CCK-8 working solution to each well, incubate shielded from light at 37°C for 1-4 hours, and measure the absorbance at 450 nm using a microplate reader.

### Immunofluorescence (IF) staining

The operation was performed as previously described. Briefly, the primary antibody Fstl1 (1:1000, ab223287, Abcam, UK) was incubated overnight at 4 ℃. Then the cells were incubated with a fluorophore-conjugated secondary antibody for 1 h and counterstained with DAPI in the dark for 5 min. Images were acquired on a laser-scanning confocal microscope.

### Cell live/dead staining

The Calcein-AM/PI live/dead cell double staining kit (BB-4126, BestBio) was used for a living and dead cell level analysis^24^. In brief, ATDC5 cells were inoculated in a 24-well plate (2 × 10^4^ cells/well). Then, different groups of cells were treated and incubated for 48 h. After washing with PBS, the cells were stained in a mixture of Calcein-AM (1: 1000) and PI (1: 2000). Finally, the state of the cells was observed using fluorescence microscopy.

### ROS staining

ROS analysis was accomplished by staining with CM-H_2_DCFDA (S0035S, Beyotime). The final concentration of CM-H_2_DCFDA was 5 μM. Cells were cultured as described above, then treated with CM-H_2_DCFDA and Hoechst 33342 for approximately 30 minutes. Subsequently, cell fluorescence was detected by fluorescence microscope (Olympus) and flow cytometry respectively (Sony ID7000).

### Mitochondrial fluorescent staining (JC-1, Mito-Tracker, MitoSOX and mPTP)

Details of the method for JC-1, Mito-Tracker, and MitoSOX experiments have been previously described. Briefly, the JC-1 experiment was performed using an enhanced mitochondrial membrane potential detection kit (JC-1) (C2003S, Beyotime, China) following standardized protocols. Mito-Tracker experiment was performed using Mito-Tracker Red CMXRos (C1035, Beyotime, China) following standardized protocols. MitoSOX experiment was performed using MitoSOX Red (S0061S; Beyotime, China) following standardized protocols. For mPTP staining, it was detected by an mPTP Assay Kit (C2009S, Beyotime, China). Briefly, the treated cells were washed with PBS and incubated with calcein AM plus Co^2+^ quencher at 37 °C for 30 min. Then, the dye was replaced by culture medium, and the slides were cultured at 37 °C for 30 min in the dark and observed by a fluorescence microscope^25^.

### Transmission electron microscope (TEM) experiments

Details of the method for TEM experiments have been previously described. The samples were ultra-thin sectioned (50-100 nm) using an ultra-micro microtome. High-resolution imaging was performed using TEM system (H-7650, Hitachi, Tokyo, Japan) to observe the mitochondrial microstructure and ultrastructural details of the samples.

### Construction and characterization of C-LNP^@Fstl1^

DLin-MC3-DMA (MC3), cholesterol, DSPC, PEG2000, and DSPE-PEG-NHS were individually dissolved in anhydrous ethanol at equivalent molar concentrations of 100 mM, 10 mM, 10 mM, 10 mM, and 10 mM, respectively, forming a lipid-ethanol phase solution. Concurrently, Fstl1-shRNA was diluted in citrate buffer to prepare an aqueous nucleic acid phase solution (1 μg/μL). Employing a microfluidic device, the lipid-ethanol and nucleic acid-citrate phases were mixed at a 3:1 volumetric ratio to synthesize LNP^@Fstl1^. Subsequent surface modification was achieved through stable amide bond formation between NHS groups of DSPE-PEG-NHS and primary amine groups of CAP, yielding cartilage-targeting C-LNP^@Fstl1^.

The particle size, morphology, and zeta potential of LNP^@Fstl1^ and C-LNP^@Fstl1^ were analyzed using TEM (H-7650, Hitachi, Tokyo, Japan) and DLS (Malvern Instruments Ltd., Malvern, UK). C-LNP^@Fstl1^ was resuspended in PBS and DMEM/F12 complete medium, with hydrodynamic stability of nanoparticles via DLS over 10 days at 37 °C.

### Preparation of HA-PBA

A solution of 5.856 mg 2-morpholinoethanesulfonic acid (MES) in 300 mL deionized water was adjusted to pH 5.50 ± 0.05 using 1 M NaOH under vigorous magnetic stirring, with pH monitored via calibrated digital pH meter. After adding 1.0 g hyaluronic acid (HA) and dissolving completely at 25 °C, 1.4 g 4-(4,6-dimethoxy-1,3,5-triazin-2-yl)-4-methylmorpholinium chloride (DMTMM) was introduced and stirred for 30 min, followed by reaction with 0.11 g 3-aminophenylboronic acid (3-PBA) for 24 hr at room temperature. The crude product was purified through dialysis against deionized water (14 kDa MWCO, 5 days with 6-hour interval water changes) and lyophilized to constant weight, yielding HA-PBA as a porous matrix.

### Construction and characterization of HA-PBA/TA self-healing hydrogel

HA-PBA (30.0 mg) was precisely weighed and dissolved in 1 mL PBS at 40 °C with concurrent ultrasonic oscillation until complete dissolution yielded a clear 2% (w/v) solution. Separately, tannic acid (TA, 10 mg) was dissolved in 1 mL PBS containing pre-synthesized C-LNP^@Fstl1^ via 5-minute sonication, producing a homogeneous TA solution with dispersed nanoparticles.

Prepare the HA-PBA solution and transfer it to a centrifuge tube. Under vortex oscillation conditions, rapidly add the TA solution dropwise into the aforementioned dissolution system. After 5-10 seconds of continuous vortex oscillation, the solution immediately undergoes cross-linking and solidification, resulting in HA-PBA/TA self-healing hydrogel. The dynamic cross-linking between PBA and TA through borate ester bonds enables reversible structural integrity. The cured hydrogel system can be delivered to fracture sites via syringe compression and re-crosslinks in situ to form a solidified hydrogel.

Samples of pristine HA-PBA/TA hydrogel and C-LNP^@Fstl1^-loaded hydrogel were cryo-fractured in liquid nitrogen, lyophilized, and sectioned with surgical blades to expose cross-sections. After mounting on scanning electron microscope (SEM) stubs and gold sputter-coating (90 s), their surface morphology was examined. Rheological properties of HA-PBA/TA hydrogels were quantified via DHR-2 rotational rheometer (TA Instruments, USA) equipped with a 40-mm parallel-plate geometry by sequentially performing time sweeps, steady-shear tests, amplitude sweeps, frequency sweeps, and step-strain measurements.

To evaluate the release kinetics of C-LNP^@Fstl1^ from the hydrogel matrix, samples loaded with FITC-labeled C-LNP^@Fstl1^ were incubated in 1 mL ddH₂O. A fluorescence intensity-concentration standard curve was established, and supernatants were collected at predetermined intervals to quantify fluorescence intensity, thereby calculating C-LNP^@Fstl1^ release concentrations and plotting cumulative release profiles.

### Data processing

All experiments in this study were independently repeated three or more times, and the data are presented as mean ± standard deviation (SD). Data analysis and comparisons were performed using GraphPad Prism software (version 9.5.1). For statistical analysis, a t-test was used to compare data between two groups, and a one-way analysis of variance (ANOVA) was used to analyze and compare data between multiple groups. Differences were considered statistically significant when the *P*-value was less than 0.05.

## Results

### Deletion of Piezo1 inhibits chondrocytes transdifferentiation to osteoblasts and increases inflammatory response of chondrocytes

**Figure 1:**
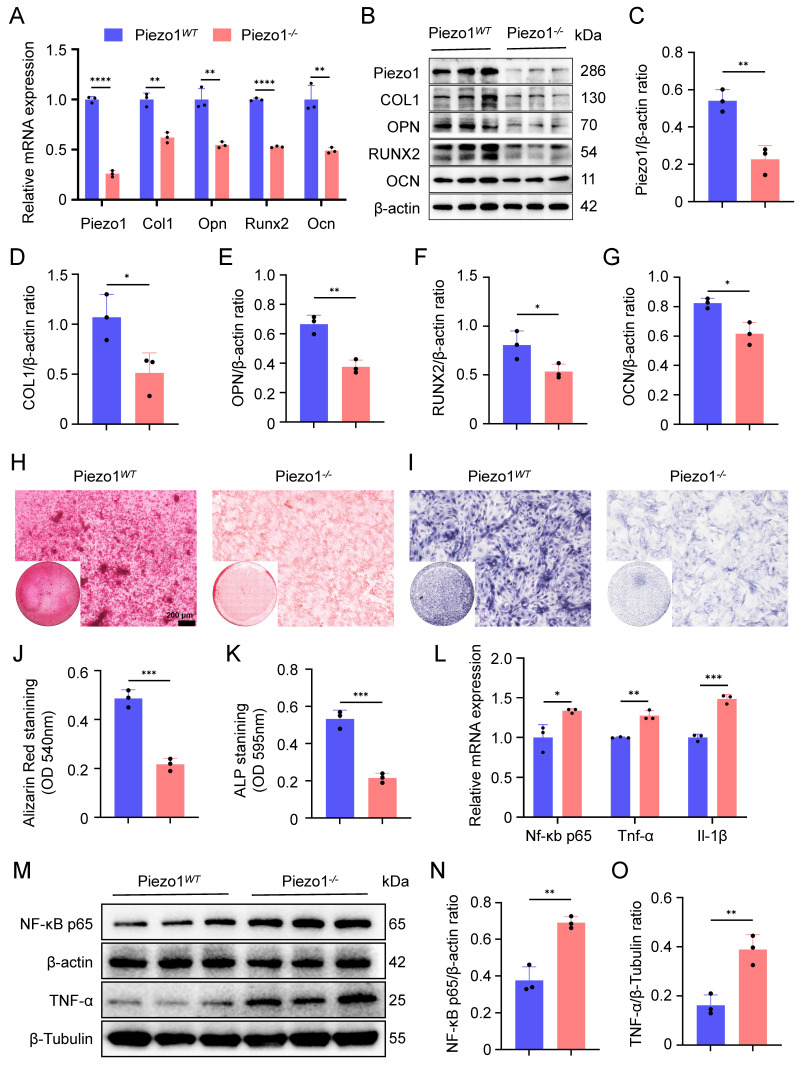
** Deletion of Piezo1 inhibits chondrocytes transdifferentiation to osteoblasts and increases inflammatory response. (A)** qPCR was used to detect changes in the expression of Piezo1 and osteogenic markers Col1, Opn, Runx2, and Ocn in each group after osteogenic induction culture (Data are represented as means ± SD, n = 3 per group, t-test was performed, **P < 0.01, and ****P < 0.0001). **(B-G)** WB was used to detect changes in the expression of Piezo1 and osteogenic markers COL1, OPN, RUNX2, and OCN in each group after osteogenic induction culture and statistical analysis (Data are represented as means ± SD, n = 3 per group, t-test was performed, *P < 0.05, and **P < 0.01). **(H, I)** Alizarin red and ALP staining were used to detect the expression of calcium nodules after 21 days of induction culture and the ALP levels after 7 days of osteogenic induction culture, scale = 200 μm. **(J, K)** Statistical analysis of Alizarin red and ALP staining were performed (Data are represented as means ± SD, n = 3 per group, t-test was performed, *P < 0.05, ***P < 0.001). **(L)** qPCR was used to detect changes in the expression of inflammatory related factors Nf-κb p65, Tnf-α, and Il-1β in each group (Data are represented as means ± SD, n = 3 per group, t-test was performed, *P < 0.05, **P < 0.01, and ***P < 0.001). **(M-O)** WB was used to detect changes in the expression of inflammatory related proteins NF-κB p65, and TNF-α in each group after osteogenic induction culture and statistical analysis (Data are represented as means ± SD, n = 3 per group, t-test was performed, **P < 0.01).

To investigate the role of Piezo1 in endochondral ossification *in vitro*, we performed osteogenic differentiation cultures of both Piezo1*^WT^* and Piezo1*^-/-^* ATDC5 cells. The knockout efficiency of Piezo1 gene was verified by qPCR and WB (Figure 1A-C). We observed a significant reduction in the proliferative capacity of Piezo1*^-/-^* ATDC5 cells (Figure S3A). Furthermore, our results demonstrated a marked decrease in the mRNA and protein levels of the osteogenesis-related markers COL1, OPN, RUNX2, and OCN following Piezo1 deficiency (Figure 1A,B,D-G). Alizarin red staining revealed a significant reduction in calcium nodule deposition in Piezo1*^-/-^* cells compared to Piezo1*^WT^* group (Figure 1H,J). Similarly, ALP staining revealed a substantial decrease in ALP expression in Piezo1*^-/-^* cells (Figure 1I,K). Moreover, we found that compared with Piezo1*^WT^* cells, the expression of inflammatory related factors NF-κB p65, TNF-α, and IL-1β were significantly increased in Piezo1*^-/-^* cells (Figure 1L-O). And ELISA confirmed increased TNF-α protein levels in the conditioned medium in Piezo1*^-/-^* cells (Figure S3B). Collectively, these results demonstrate that Piezo1 plays a crucial role in endochondral ossification *in vitro,* its deficiency inhibits the transdifferentiation of chondrocytes into osteoblasts and increases the inflammatory factors expression.

### Specific deletion of Piezo1 in chondrocytes increased inflammatory factors expression in the callus and inhibited fracture healing

**Figure 2:**
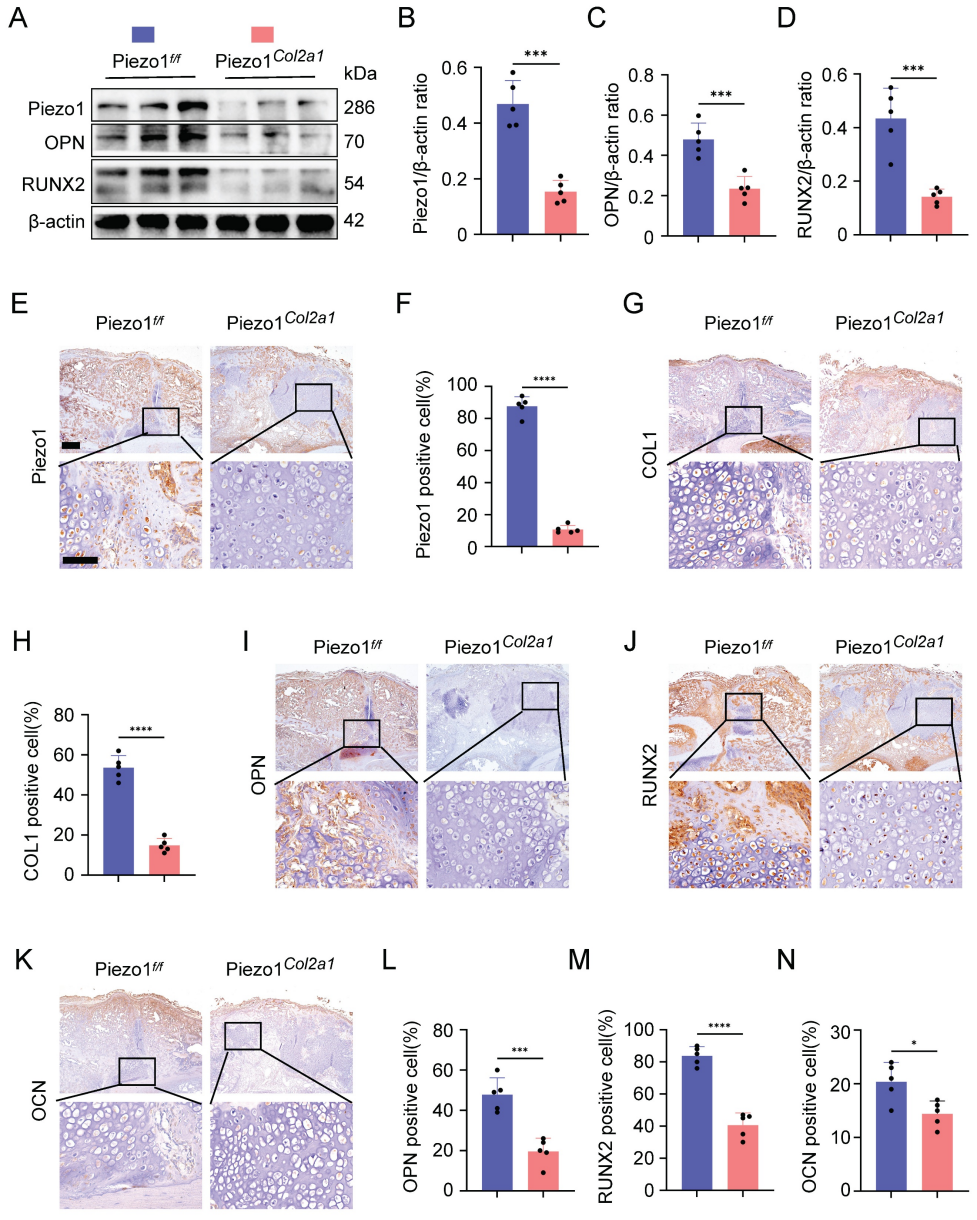
** Piezo1*^Col2a1^* mice have impaired endochondral ossification. (A-D)** WB was used to detect the expression changes of Piezo1 and osteogenic markers OPN, and RUNX2 in the callus of each group 14 days after femoral fracture, along with statistical analysis (Data are represented as means ± SD, n = 5 mice per group, t-test was performed, ***P < 0.001). **(E-N)** IHC staining was used to detect the expression changes of Piezo1 and osteogenic markers COL1, OPN, RUNX2, and OCN in hypertrophic chondrocytes of callus in each group 14 days after femoral fracture, with statistical analysis, scale = 500 μm (up) and 200 μm (down) (Data are represented as means ± SD, n = 5 mice per group, t-test was performed, *P < 0.05, ***P < 0.001, and ****P < 0.0001).

To investigate the effect of Piezo1 in chondrocytes on fracture healing, we constructed fracture models of Piezo1*^Col2a1^* mice and Piezo1 flox (Piezo1*^f/f^*) mice. The knockout efficiency of Piezo1 in the chondrocytes were verified by WB and immunohistochemistry (IHC) (Figure 2A,B,E,F). Moreover, the protein levels of osteogenesis-related markers OPN and RUNX2 were also significantly reduced in the callus of Piezo1*^Col2a1^* mice (Figure 2A,C,D). Similarly, IHC experiments revealed an increased proportion of hypertrophic chondrocytes (HTCs) in the callus of Piezo1*^Col2a1^* mice compared with Piezo1*^f/f^* mice. The expression of the osteogenic markers COL1, OPN, RUNX2, and OCN were significantly reduced in chondrocytes (Figure 2G-N).

**Figure 3:**
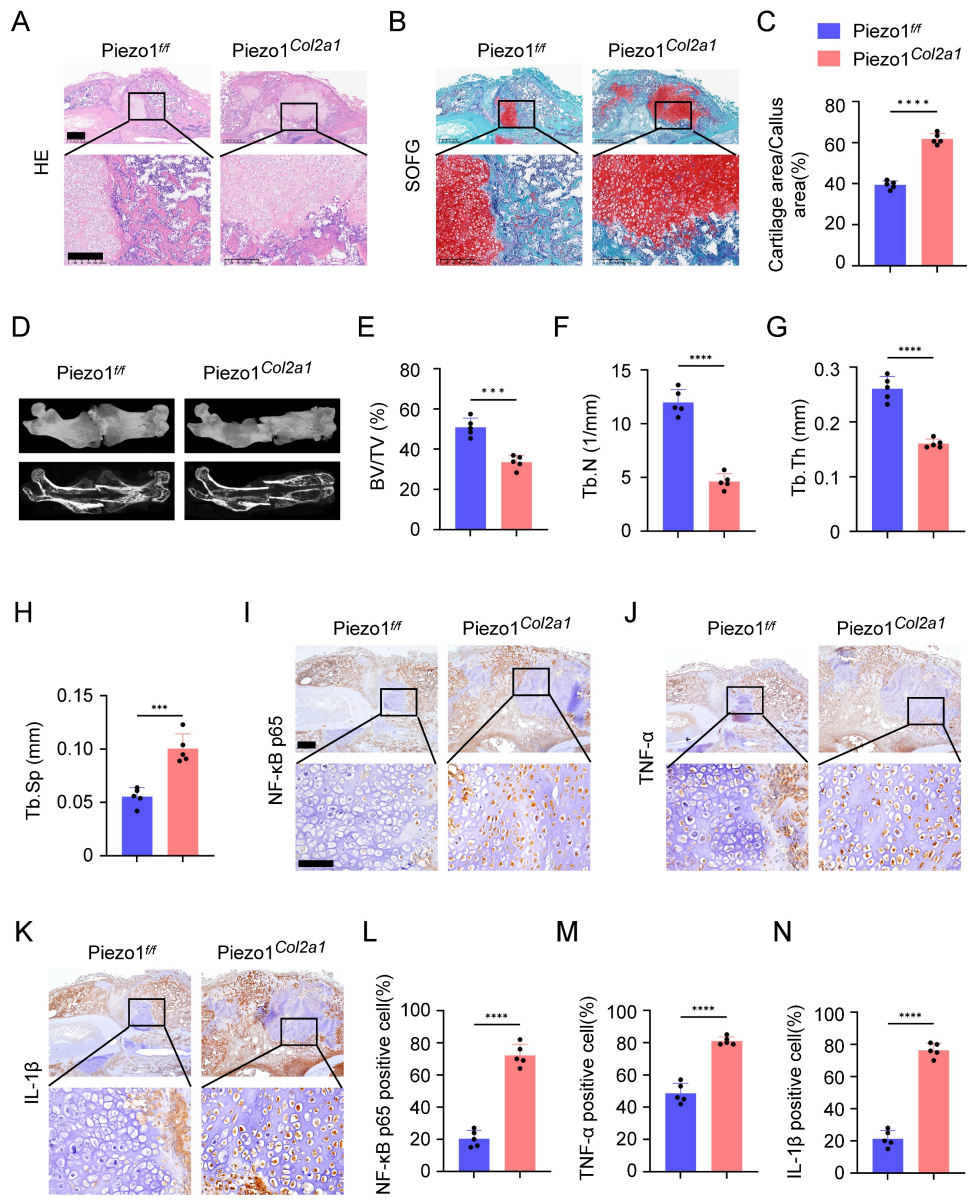
** Specific deletion of Piezo1 in chondrocytes increased inflammatory factors expression in the callus and inhibited fracture healing. (A, B)** HE and SO/FG staining were used to observe the morphological distribution of cartilage tissue and woven bone tissue in the callus of each group 14 days after femoral fracture, scale = 500 μm (up) and 200 μm (down). **(C)** The statistical analysis of the proportion of cartilage tissue to callus (Data are represented as means ± SD, n = 5 mice per group, t-test was performed, ****P < 0.0001). **(D)** Micro-CT observation of representative bone callus 3D reconstruction and coronal section images in each group 14 days after femoral fracture. **(E-H)** BV/TV, Tb.N, Tb.Th, and Tb.Sp analysis of callus 14 days after femoral fracture in each group (Data are represented as means ± SD, n = 5 mice per group, t-test was performed, ***P < 0.001, and ****P < 0.0001). **(I-N)** IHC staining was used to detect the expression changes of inflammatory related proteins NF-κB p65, TNF-α, and IL-1β in hypertrophic chondrocytes of callus in each group 14 days after femoral fracture, with statistical analysis, scale = 500 μm (up) and 200 μm (down) (Data are represented as means ± SD, n = 5 mice per group, t-test was performed, *P < 0.05, and ****P < 0.0001).

Haematoxylin-eosin (HE) staining was performed to observe morphological changes in the callus of Piezo1*^f/f^* and Piezo1*^Col2a1^* mice. Our results showed the woven bone was mainly distributed at both ends, while cartilage tissue was localised in the center, indicating that the endochondral ossification process during fracture healing started at the ends of the fracture and gradually progressed toward the center (Figure 3A). Safranin O/Fast Green (SO/FG) staining was performed to assess the morphological distribution and proportion of cartilage and woven bone tissue in the two groups of mice. The results reported that the proportion of cartilage tissue was significantly higher in Piezo1*^Col2a1^* mice than in Piezo1*^f/f^* mice (Figure 3B,C). Furthermore, we performed micro-computed tomography (micro-CT) scans on both Piezo1*^f/f^* and Piezo1*^Col2a1^* mice 14 days after femoral fracture. The results depicted a significant reduction in bony callus tissue in the Piezo1*^Col2a1^* mice (Figure 3D). BV/TV, Tb.N, and Tb.Th values of callus tissue in Piezo1*^Col2a1^* mice were significantly lower compared with Piezo1*^f/f^* mice (Figure 3E-G), while the Tb.Sp was increased (Figure 3H). Moreover, IHC experiments revealed the expression of the inflammatory factors NF-κB p65, TNF-α, and IL-1β were significantly increased in the callus of Piezo1*^Col2a1^* mice compared with Piezo1*^f/f^* mice (Figure 3I-N). These findings suggest that Piezo1 plays a crucial role in regulating cartilage-to-bone turnover as well as fracture healing.

### Inflammatory response induced chondrocyte ROS increase, mitochondrial oxidative stress and dysfunction, and hinder endochondral ossification

To investigate the effect of inflammatory factors on chondrocyte transdifferentiation to osteoblasts, we induced inflammatory response in chondrocytes with lipopolysaccharide (LPS). First, we determined the optimal concentration of LPS was 80 ng/mL by cytotoxicity test using CCK-8 kit (Figure S1A). The results showed that compared with the control group, osteogenesis related markers COL1, OPN, RUNX2, and OCN were significantly reduced in the LPS inflammation induced group (Figure S1B-G). Alizarin red and ALP staining revealed a significant reduction in calcium nodule deposition and ALP expression in LPS group (Figure S1H-K). Furthermore, we found that the expression of ROS in LPS inflammatory induced group cells was significantly increased (Figure S1L-N).

We further examined the effects of inflammation on mitochondrial function in chondrocytes. We found that LPS-induced inflammatory cells exhibited significantly reduced MMP and activity, but increased mitochondrial superoxide production (Figure S2A-F). Additionally, mPTP assay kit results demonstrated that mPTP in the LPS group were in an opening state (Figure S2G,H). And LPS-induced inflammation significantly reduced ATP levels in mitochondria (Figure S3C). Furthermore, qPCR and WB results demonstrated a marked changes in the mRNA and protein levels of the mitochondrial dynamics-related markers MFN1, MFN2, OPA1, and DRP1 in LPS inflammatory induced group (Figure S2I-N). Then, we used TEM to observe the ultrastructure of mitochondria, and found that the mitochondrial matrix was enlarged, the mitochondrial crest was reduced or disappeared, and the inner and outer membranes of mitochondria were incomplete and damaged in LPS group (Figure S2O). These results suggest that inflammatory response can induce ROS increase, mitochondrial oxidative stress, mPTP opening and dysfunction in chondrocytes and hinder endochondral ossification *in vitro.*

### Activation of Piezo1 can reduce inflammation and promote endochondral ossification

To further assess whether Piezo1 activation alleviates inflammation and promotes endochondral ossification, we conducted intervention experiments using the Piezo1-specific agonist Yoda1. qPCR and WB analyses revealed that Yoda1 significantly reduced the expression of inflammatory factors NF-κB p65 and TNF-α in ATDC5 cells (Figure S3F-H), and ELISA confirmed decreased TNF-α protein levels in the conditioned medium (Figure S3I). Additionally, detection of osteogenic markers showed that Yoda1 treatment markedly increased the expression of COL1, OPN, RUNX2, and OCN in chondrocytes (Figure S3K,L). These results demonstrate that Piezo1 activation can mitigate inflammatory responses and enhance endochondral ossification.

### Piezo1 could regulate Fstl1 expression both *in vivo* and *in vitro*

**Figure 4:**
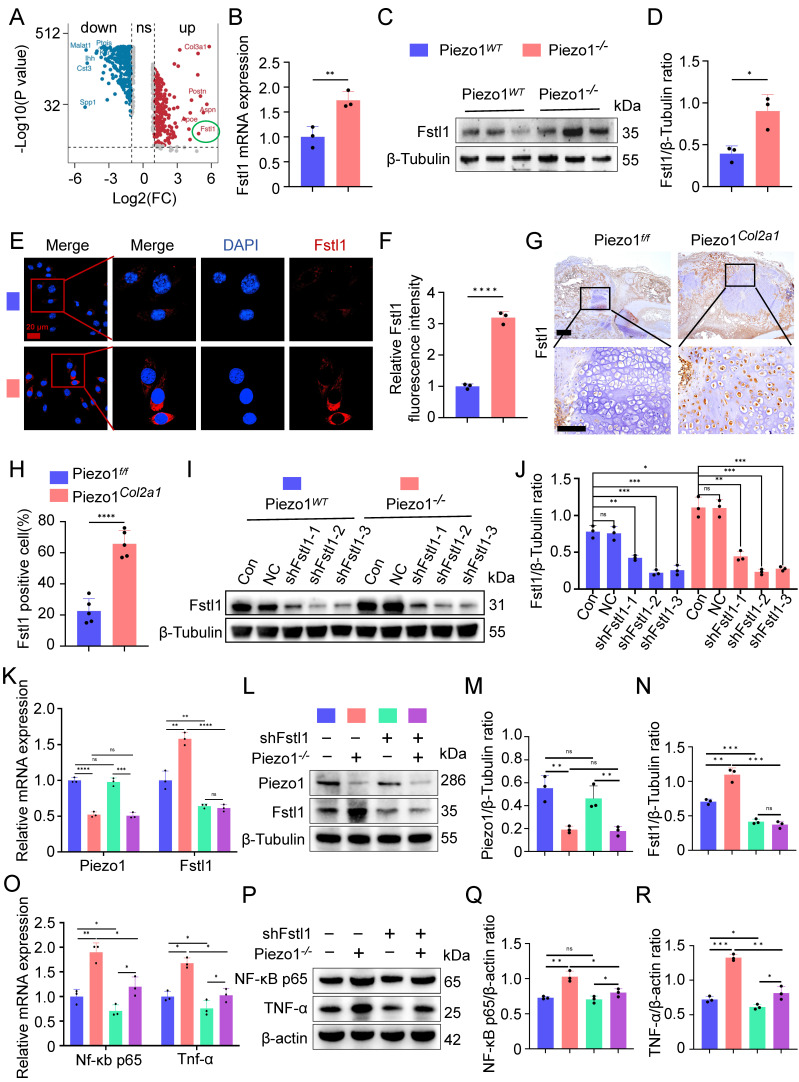
** Piezo1 could regulate Fstl1 expression both *in vivo* and *in vitro*, and shFstl1 plasmids were constructed. (A)** Differentially expressed genes in hypertrophic chondrocytes after specific knockout of Piezo1 in mouse chondrocytes. **(B)** qPCR was performed to detect changes in Fstl1 expression in each group (Data are represented as means ± SD, n = 3 per group, t-test was performed, **P < 0.01). **(C, D)** WB was used to detect changes in Fstl1 expression in each group and statistical analysis (Data are represented as means ± SD, n = 3 per group, t-test was performed, *P < 0.05). **(E, F)** IF staining was used to detect changes in Fstl1 expression in each group and statistical analysis, scale = 20 μm (Data are represented as means ± SD, n = 3 per group, t-test was performed, ****P < 0.0001). Blue fluorescence indicates the nucleus, and red fluorescence indicates the Fstl1 protein. **(G, H)** IHC staining was used to detect the expression changes of Fstl1 in hypertrophic chondrocytes of callus in each group 14 days after femoral fracture and statistical analysis, scale = 500 μm (up) and 200 μm (down) (Data are represented as means ± SD, n = 5 mice per group, t-test was performed, ****P < 0.0001). **(I, J)** WB was used to determine the knockdown efficiency of shFstl1 interference plasmids transfected with different sequences in each group, and statistical analysis was performed (Data are represented as means ± SD, n = 3 per group, ANOVA was performed to compare data between groups, *P < 0.05, **P < 0.01, ***P < 0.001, and ns > 0.05). **(K)** qPCR was performed to detect changes in Piezo1 and Fstl1 expression in each group (Data are represented as means ± SD, n = 3 per group, ANOVA was performed to compare data between groups, **P < 0.01, ***P < 0.001, ****P < 0.0001, and ns > 0.05). **(L-N)** WB was used to detect changes in Piezo1 and Fstl1 expression in each group and statistical analysis (Data are represented as means ± SD, n = 3 per group, ANOVA was performed to compare data between groups, **P < 0.01, ***P < 0.001, and ns > 0.05). **(O)** qPCR was performed to detect changes in Nf-κb p65, and Tnf-α expression in each group (Data are represented as means ± SD, n = 3 per group, ANOVA was performed to compare data between groups, *P < 0.05, and **P < 0.01). **(P-R)** WB was used to detect changes in NF-κB p65, and TNF-α expression in each group and statistical analysis (Data are represented as means ± SD, n = 3 per group, ANOVA was performed to compare data between groups, *P < 0.05, **P < 0.01, ***P < 0.001, and ns > 0.05).

To further explore how Piezo1 regulates inflammatory response and affects the process of endochondral ossification in fracture healing, we analysed scRNA-seq data from the callus tissues of Piezo1*^f/f^* and Piezo1*^Col2a1^* mice 14 days after femoral fracture. From callus tissues, we collected 27,593 single cells for analysis. Based on marker gene expression, we annotated cell types and identified nine distinct clusters. These included stromal cells, pericytes, endothelial cells, and immune cell populations such as hematopoietic stem/progenitor cells (HSPCs), neutrophils, monocytes, macrophages, osteoclasts, and lymphocytes. Detailed analysis of scRNA-seq data is presented in a previously published article^22^. We examined the differentially expressed genes (DEGs) in HTCs of Piezo1*^Col2a1^* and Piezo1*^f/f^* groups and found that Fstl1 was significantly upregulated in chondrocytes after Piezo1 deficiency (Figure 4A). Fstl1, a novel pro-inflammatory protein, which can promote the inflammatory response of chondrocytes through NF-κB signaling pathway and aggravate the progression of arthritis^26^.

To verify the results of scRNA-seq, we performed a series of experiments *in vivo* and *in vitro*. qPCR and WB analyses revealed that both the mRNA and protein levels of Fstl1 were significantly elevated in Piezo1*^-/-^* cells compared with the Piezo1*^WT^* group (Figure 4B-D). Immunofluorescence staining further confirmed the markedly increased expression of Fstl1 in Piezo1*^-/-^* cells (Figure 4E,F). Activation of Piezo1 with Yoda1 significantly reduced Fstl1 expression (Figure S3G,H). Then we measured the baseline levels of Fstl1 and found that its expression was minimal in non-fracture tissues (Figure S3M). Furthermore, IHC revealed significantly increased Fstl1 expression in chondrocytes of callus tissue from Piezo1*^Col2a1^* mice compared to the Piezo1*^f/f^* mice(Figure 4G,H). Collectively, these findings suggest that Piezo1 deficiency leads to an increase in Fstl1 levels both *in vitro* and *in vivo*.

### Inhibition of Fstl1 reduced the inflammatory response and ROS expression in chondrocytes, ameliorated mitochondrial function and endochondral ossification

To investigate whether Fstl1 acts as a key downstream regulator of Piezo1, we constructed Fstl1 interference plasmids (shFstl1) and a negative control plasmid (NC) for *in vitro* experiments. Three interference sequences targeting Fstl1 were designed to construct corresponding plasmids (shFstl1-1, shFstl1-2, and shFstl1-3) (Table S2). The results indicated that shFstl1-2 plasmid demonstrated the most effective interference (Figure 4I,J). Therefore, shFstl1-2 interference plasmid was selected for subsequent studies. Then the plasmid was transfected into both Piezo1*^WT^* and Piezo1*^-/-^* cells. qPCR and WB results showed that our Piezo1*^-/-^* cells construction and shFstl1 plasmid transfection were successful (Figure 4K-N).

**Figure 5:**
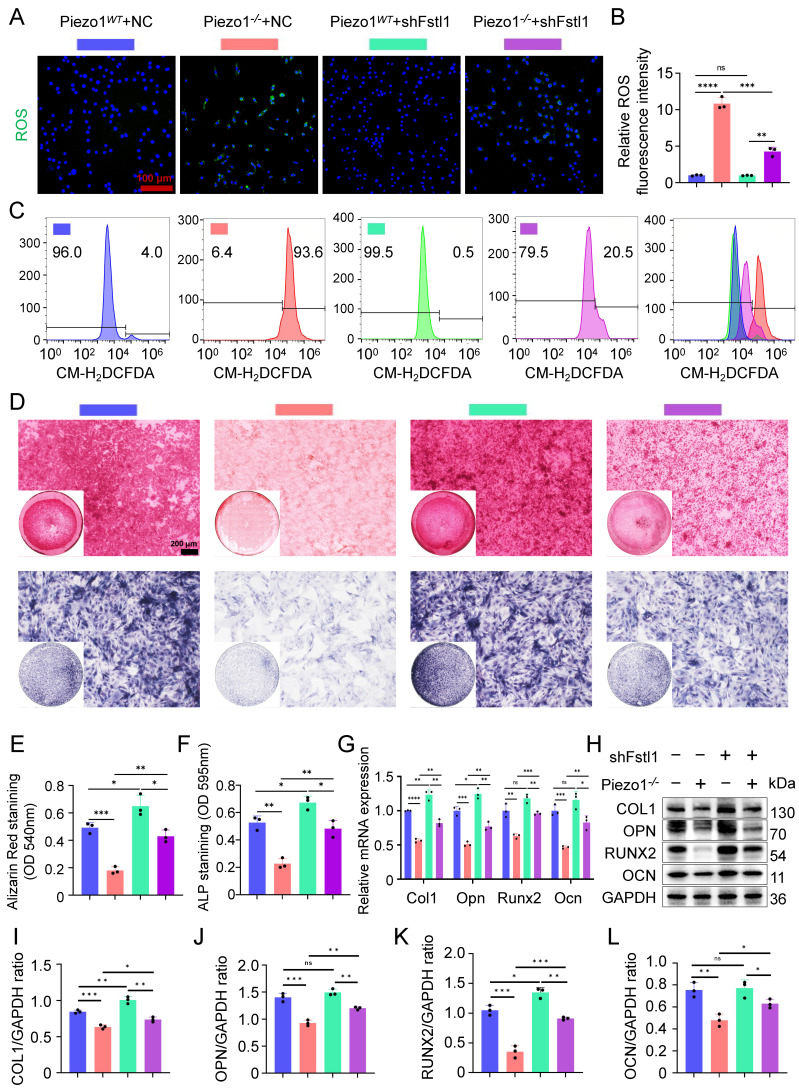
** Inhibition of Fstl1 reduced the inflammatory response and ROS expression in chondrocytes, ameliorated endochondral ossification. (A, B)** ROS staining was used to detect the fluorescence intensity of CM-H2DCFDA in each group, and statistical analysis was performed, scale = 100 μm (Data are represented as means ± SD, n = 3 per group, ANOVA was performed to compare data between groups, ***P < 0.001, ****P < 0.0001, and ns > 0.05). **(C)** Representative flow cytometry image of mean fluorescence intensity of ROS in each group. **(D)** Alizarin red and ALP staining were used to detect the expression of calcium nodules after 21 days of induction culture and the ALP levels after 7 days of osteogenic induction culture, scale = 200 μm. **(E, F)** Statistical analysis of Alizarin red and ALP staining were performed (Data are represented as means ± SD, n = 3 per group, ANOVA was performed to compare data between groups, *P < 0.05, **P < 0.01, and ***P < 0.001). **(G)** qPCR was used to detect changes in the expression of osteogenic markers Col1, Opn, Runx2, and Ocn in each group after osteogenic induction culture (Data are represented as means ± SD, n = 3 per group, ANOVA was performed to compare data between groups, *P < 0.05, **P < 0.01, ***P < 0.001, ****P < 0.0001, and ns > 0.05). **(H-L)** WB was used to detect changes in the expression of osteogenic markers COL1, OPN, RUNX2, and OCN in each group after osteogenic induction culture and statistical analysis (Data are represented as means ± SD, n = 3 per group, ANOVA was performed to compare data between groups, *P < 0.05, **P < 0.01, ***P < 0.001, and ns > 0.05).

We found that the expression of inflammatory related factors NF-κB p65, and TNF-α in the mRNA and protein levels were significantly reduced in Piezo1*^-/-^* cells after Fstl1 was inhibited (Figure 4O-R). ROS staining and flow cytometry results showed that inhibition of Fstl1 significantly reduced the fluorescence intensity of ROS in Piezo1*^-/-^* cells (Figure 5A-C). Furthermore, inhibition of Fstl1 rescued impaired endochondral ossification in Piezo1*^-/-^* cells, as assessed by increased osteogenesis-related markers COL1, OPN, RUNX2, and OCN (Figure 5G-L). The alizarin red and ALP staining results support these findings; however, the calcification does not fully return to control levels after Fstl1 knockdown (Figure 5D-F). This may involve other potential regulatory factors and parallel pathways. To this end, we inhibited the NF-κB signaling pathway using JSH-23. The results showed that NF-κB inhibition promoted the expression of osteogenic genes COL1, RUNX2, and OCN in Piezo1*^-/-^* cells, indicating that NF-κB is a downstream signaling pathway of Piezo1 (Figure S3D,E).

**Figure 6:**
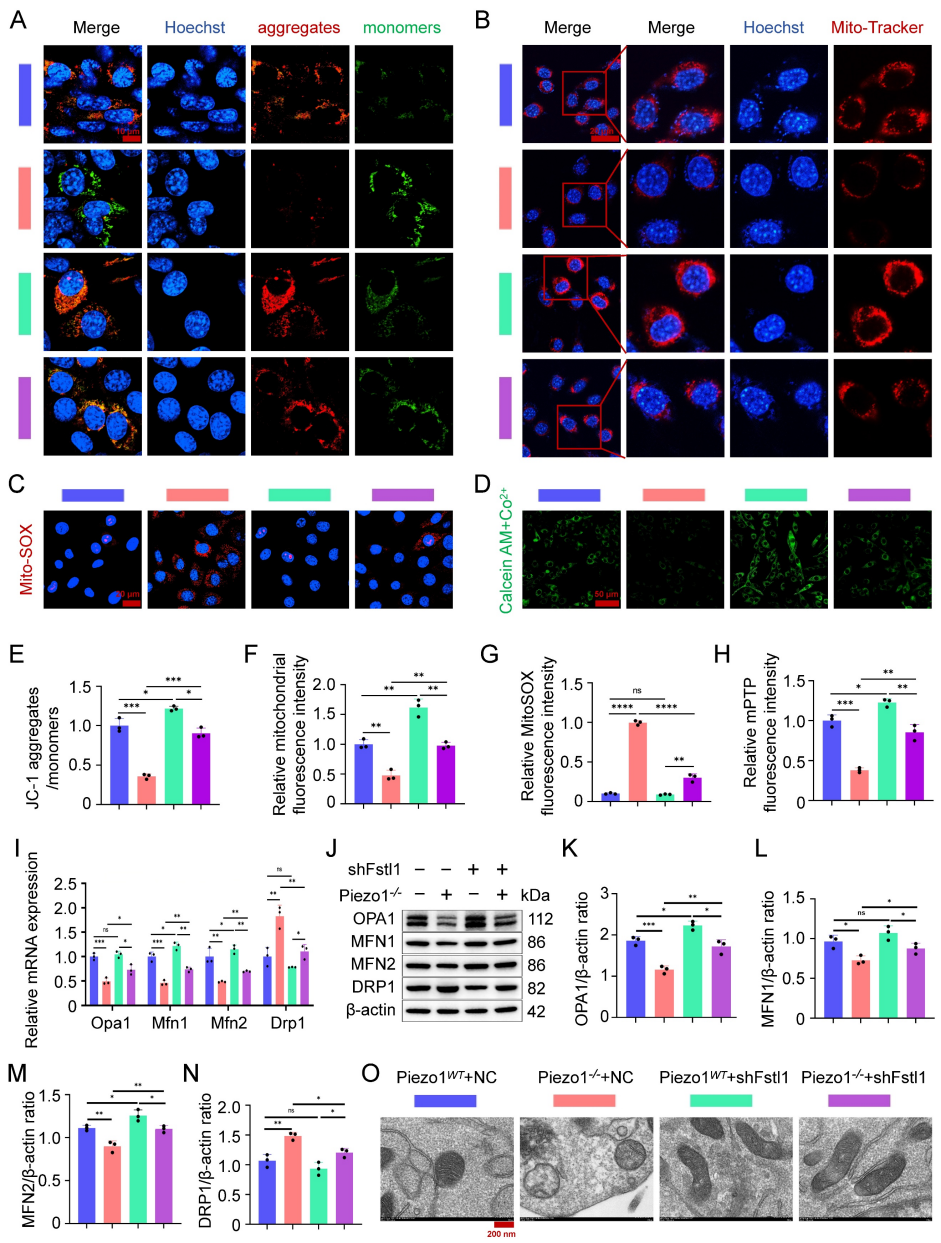
** Inhibition of Fstl1 ameliorated mitochondrial oxidative stress and dysfunction. (A)** JC-1 staining was used to detect changes in mitochondrial membrane potential in each group, scale = 10 μm. **(B)** Mito-Tracker Red staining was used to detect fluorescence changes in biologically active mitochondria in each group, scale = 20 μm. **(C)** MitoSOX Red was used to detect changes in the expression of superoxide in the mitochondria of each group, scale = 20 μm. **(D)** mPTP Assay Kit was used to detect changes in the expression of Calcein green fluorescence in the mitochondria of each group, scale = 50 μm. **(E-H)** Statistical analysis of fluorescence intensity of JC-1, Mito-Tracker Red, MitoSOX Red, and mPTP staining (Data are represented as means ± SD, n = 3 per group, ANOVA was performed to compare data between groups, *P < 0.05, **P < 0.01, ***P < 0.001, ****P < 0.0001, and ns > 0.05). **(I)** qPCR was used to detect the expression of mitochondrial dynamics-related genes (Opa1, Mfn1, Mfn2, and Drp1) in each group (Data are represented as means ± SD, n = 3 per group, ANOVA was performed to compare data between groups, *P < 0.05, **P < 0.01, ***P < 0.001, and ns > 0.05). **(J-N)** WB was used to detect changes in the expression of mitochondrial dynamics-related markers (OPA1, MFN1, MFN2, and DRP1) in each group, and statistical analysis (Data are represented as means ± SD, n = 3 per group, ANOVA was performed to compare data between groups, *P < 0.05, **P < 0.01, ***P < 0.001, and ns > 0.05). **(O)** TEM was used to observe the mitochondrial microstructure and ultrafine details of the cells in each group, scale = 200 nm, n = 3 per group.

Fstl1 deficiency increased MMP and mitochondrial activity and decreased mitochondrial superoxide synthesis in Piezo1^-/-^ cells, as measured by JC-1 staining (Figure 6A,E), Mito-Tracker Red staining (Figure 6B,F), and MitoSOX Red staining (Figure 6C,G). In addition, we found that inhibition of Fstl1 enhanced the green fluorescence of Calcein in the mitochondria of Piezo1*^-/-^* cells, indicating that mPTP closure was restored (Figure 6D,H). And inhibition of Fstl1 in Piezo1*^-/-^* cells modulated mitochondrial dynamics-related markers OPA1, MFN1, MFN2, and DRP1, and then restored mitochondrial functional homeostasis (Figure 6I,N). Similarly, we observed the ultrastructure of mitochondria by TEM and found that after Fstl1 inhibition, mitochondrial matrix swelling was reduced and mitochondrial crest increased (Figure 6O). These results confirmed that Piezo1 regulates the inflammatory response and ROS expression of chondrocytes through the Fstl1, affects mitochondrial oxidative stress and dysfunction, and then regulates endochondral ossification.

### Construction and characterization of C-LNP^@Fstl1^ and HA-PBA/TA self-healing hydrogels

**Figure 7:**
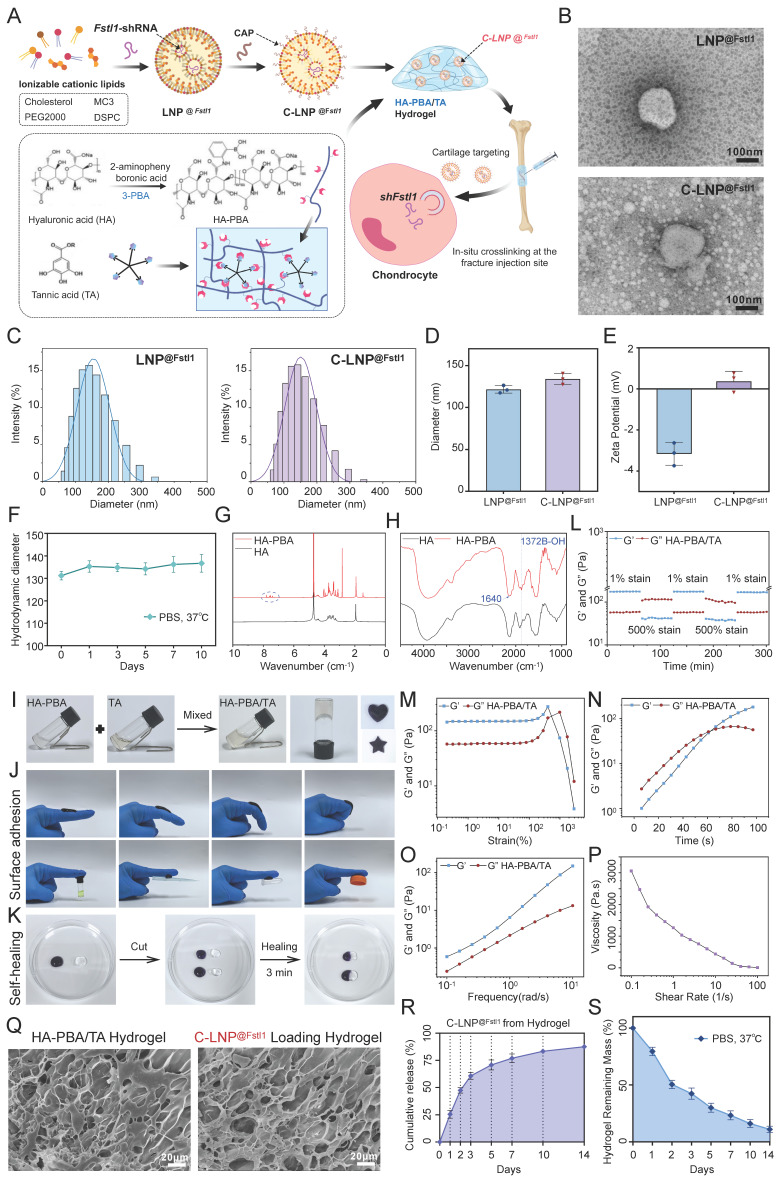
** Construction and characterization of C-LNP^@Fstl1^ and HA-PBA/TA self-healing hydrogels. (A)** Schematic diagram of synthesis of C-LNP^@Fstl1^ and HA-PBA/TA self-healing hydrogels. **(B)** Electron microscopy images of LNP^@Fstl1^ and C-LNP^@Fstl1^, scale = 100 nm, n = 3 per group. **(C, D)** DLS was used to detect the particle size distribution and average particle size of LNP^@Fstl1^ and C-LNP^@Fstl1^ (Data are represented as means ± SD, n = 3 per group, t-test was performed). **(E)** DLS was used to detect the zeta potential of the surfaces of LNP^@Fstl1^ and C-LNP^@Fstl1^ (Data are represented as means ± SD, n = 3 per group, t-test was performed). **(F)** DLS was used to detect the structural stability of C-LNP^@Fstl1^. **(G)** ¹H NMR analysis confirmed that HA and PBA were successfully grafted. **(H)** FT-IR analysis showed that PBA was successfully grafted on HA. **(I)** Schematic diagram of HA-PBA/TA self-healing hydrogel gel formation and mold appearance. **(J)** Schematic diagram of hydrogel adhesion. **(K)** Schematic diagram of self-healing appearance of hydrogel. **(L-P)** Rheological performance curve of hydrogel, **(L)** step rheology, **(M)** strain rheology, **(N)** time rheology, **(O)** frequency rheology, **(P)** shear steady state. **(Q)** SEM image of HA-PBA/TA hydrogel and C-LNP^@Fstl1^ loading hydrogel, scale = 20 μm, n = 3 per group. **(R, S)** C-LNP^@Fstl1^ release and hydrogel degradation curve.

To investigate the regulatory role of Fstl1 on fracture healing in vivo, we initially synthesized lipid nanoparticles loaded with shFstl1 (LNP^@shFstl1^). Subsequent surface modification with a CAP yielded cartilage-targeting C-LNP^@shFstl1^ (Figure 7A). Furthermore, to achieve sustained and localized release of C-LNP^@shFstl1^ within the callus microenvironment, we engineered an HA-PBA/TA self-healing hydrogel platform. The C-LNP^@shFstl1^-laden hydrogel was then administered via in situ injection at fracture sites for subsequent mechanistic studies (Figure 7A). TEM revealed uniformly dispersed spherical nanostructures for both LNP^@shFstl1^ and its chondrocyte-targeting counterpart C-LNP^@shFstl1^ (Figure 7B). The particle size distribution and average particle size of LNP^@Fstl1^ and C-LNP^@Fstl1^ were detected by dynamic light scattering (DLS). The results showed that the average particle size of LNP^@Fstl1^ and C-LNP^@Fstl1^ was 121.6 nm and 133.9 nm respectively, and both groups of nanoparticles were well dispersed (Figure 7C,D). Zeta potential measurements further showed surface charges of -3.16 ± 0.58 mV for LNP^@shFstl1^ and 0.37 ± 0.38 mV for the CAP-modified C-LNP^@shFstl1^ (Figure 7E). Post-functionalization stability assessment confirmed retained structural integrity of C-LNP^@shFstl1^, exhibiting less than 15% fluctuation in particle size distribution when incubated in PBS at 37 °C over 10 days (Figure 7F).

Nuclear magnetic resonance hydrogen (¹H NMR) spectroscopy confirmed successful chemical grafting of PBA onto HA chains (Figure 7G), which was further validated by fourier transform infrared spectroscopy (FT-IR) analysis showing characteristic covalent bonding signatures (Figure 7H). Macroscopic observation demonstrated immediate formation of stable cross-linked hydrogels upon mixing HA-PBA and TA solutions (Figure 7I). The material exhibited notable moldability, maintaining structural integrity in customized shapes (Figure 7I), and demonstrated robust adhesiveness to finger surfaces at various angles and against diverse adherent substrates (Figure 7J). Remarkable self-healing capability was observed through complete structural recovery within 3 min of contact between severed interfaces, with no visible fractures post-reintegration (Figure 7K). Rheological characterization revealed the hydrogel's dynamic crosslinking behavior and self-healing capability through step-strain measurements (Figure 7L). Strain amplitude sweeps identified the linear viscoelastic region limit and structural yielding points (Figure 7M). The time rheology results showed that the crosslinking network structure of the hydrogel was stable and no obvious collapse occurred (Figure 7N). The frequency rheology results showed that the network structure of the hydrogel was stable and had good mechanical stability (Figure 7O). Steady-shear analysis validated injectability (Figure 7P). Scanning electron microscope (SEM) imaging revealed an interconnected 3D porous network optimized for drug encapsulation and sustained release, with discrete nanostructure inclusions confirming successful C-LNP^@shFstl1^ encapsulation (Figure 7Q). We further analyzed the correlation between the degradation kinetics of the HA-PBA/TA hydrogel and the release behavior of C-LNP^@Fstl1^ in the revised manuscript. The results revealed a strong negative correlation (R² = 0.9806) between the cumulative release of LNPs and the remaining mass percentage of the hydrogel (Figure S3O). Finally, we detected the release of C-LNP^@Fstl1^ (Figure 7R) and the degradation profiles of hydrogel (Figure 7S), then verified the in vivo applicability of this therapeutic system.

### The HA-PBA/TA self-healing hydrogel loaded with C-LNP^@Fstl1^ significantly reduced inflammatory cytokine in callus tissue and accelerated fracture healing

Initially, we evaluated the cytotoxicity of C-LNP^@Fstl1^, HA-PBA/TA hydrogel, and C-LNP^@Fstl1^ hydrogel using live/dead cell staining. Results demonstrated no significant adverse effects on cell viability (Figure S4A,B). Subsequently, the hydrogel constructs were administered via in situ injection at murine fracture sites (Figure S4C). To verify its targeting effect on chondrocytes, we introduced cartilage-targeting lipid nanoparticles labeled with green fluorescent protein (GFP-C-LNP^@Fstl1^) and performed tissue fluorescence staining to validate their targeted accumulation in chondrocytes within the callus. The green signal (GFP-C-LNP^@Fstl1^) extensively overlapped with the blue signal (nuclei of chondrocytes), demonstrating highly specific targeting of chondrocytes in the callus (Figure S3N). HE staining of major visceral organs (heart, liver, spleen, lung, and kidney) revealed no significant histological differences or pathological alterations across experimental groups, indicating favorable biosafety profiles of the material (Figure S4D).

**Figure 8:**
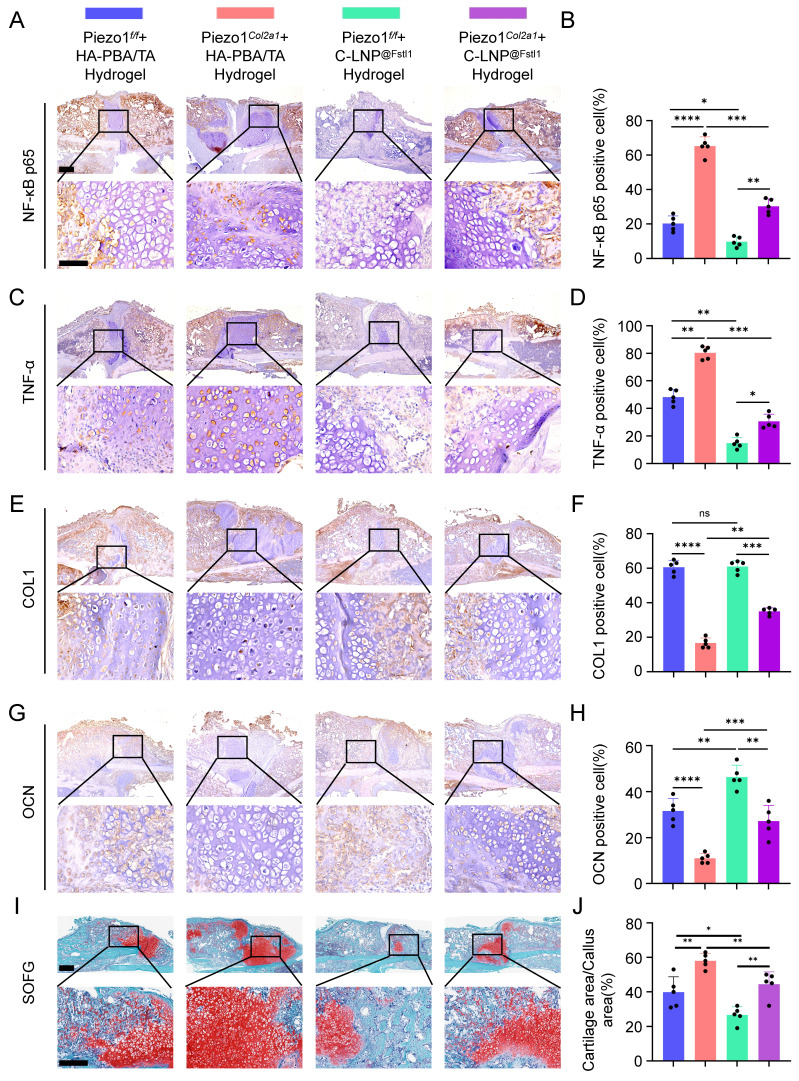
** The HA-PBA/TA self-healing hydrogel loaded with C-LNP^@Fstl1^ reduced inflammatory cytokine and increased osteogenic factors in callus tissue. (A-D)** IHC staining was used to detect the expression of inflammatory related proteins NF-κB p65, and TNF-α in hypertrophic chondrocytes of callus in each group 14 days after femoral fracture, with statistical analysis, scale = 500 μm (up) and 200 μm (down); (Data are represented as means ± SD, n = 5 mice per group, ANOVA was performed to compare data between groups, *P < 0.05, **P < 0.01, ***P < 0.001, and ****P < 0.0001). **(E-H)** IHC staining was used to detect the expression of osteogenic markers COL1, and OCN in hypertrophic chondrocytes of callus in each group 14 days after femoral fracture, with statistical analysis; (Data are represented as means ± SD, n = 5 mice per group, ANOVA was performed to compare data between groups, **P < 0.01, ***P < 0.001, ****P < 0.0001, and ns > 0.05). **(I, J)** SO/FG staining and statistical analysis were performed to determine the proportion of cartilage tissue to callus, scale = 500 μm (up) and 200 μm (down); (Data are represented as means ± SD, n = 5 mice per group, ANOVA was performed to compare data between groups, *P < 0.05, and **P < 0.01).

**Figure 9:**
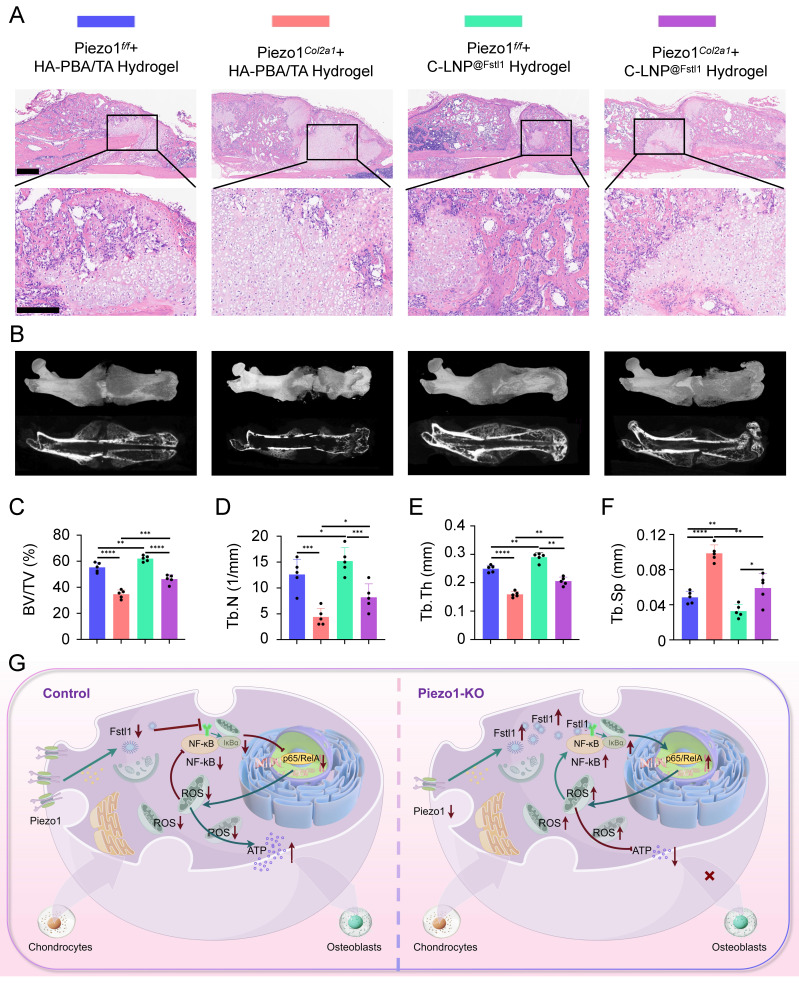
** The HA-PBA/TA self-healing hydrogel loaded with C-LNP^@Fstl1^ accelerated fracture healing. (A)** HE staining was used to observe the morphological distribution of cartilage tissue and woven bone tissue in the callus of each group 14 days after femoral fracture, scale = 500 μm (up) and 200 μm (down), n = 5 mice per group. **(B)** Micro-CT observation of representative bone callus 3D reconstruction and coronal section images in each group 14 days after femoral fracture, n = 5 mice per group. **(C-F)** BV/TV, Tb.N, Tb.Th, and Tb.Sp analysis of callus 14 days after femoral fracture in each group (Data are represented as means ± SD, n = 5 mice per group, ANOVA was performed to compare data between groups, *P < 0.05, **P < 0.01, ***P < 0.001, and ****P < 0.0001). **(G)** Schematic diagram of molecular mechanism of this study.

The knockout efficiency of Piezo1 and Fstl1 in the chondrocytes were verified by IHC staining (Figure S5A-D). And the expression of inflammatory factors NF-κB p65, TNF-α, and IL-1β in femoral fractures were examined in each treated group by IHC staining (Figure 8A-D; Figure S5E,F). The findings revealed that C-LNP^@Fstl1^ Hydrogel treatment reduced inflammatory cytokine in the callus of Piezo1*^Col2a1^* mice. Moreover, the expression of osteogenic markers COL1, OPN, RUNX2, and OCN in femoral fractures were examined in each treated group by IHC staining (Figure 8E-H; Figure S5G-J). SO/FG staining and statistical analysis were performed to determine the proportion of chondrocytes in the callus (Figure 8I-J). The proportion of chondrocytes in the C-LNP^@Fstl1^ Hydrogel group was significantly lower than that in the blank hydrogels group, indicating that the inhibition of elevated levels of Fstl1 in chondrocytes promotes endochondral ossification and accelerates fracture healing. And the HE staining results support these findings (Figure 9A). Finally, we performed micro-CT scans of the mouse specimens 14 days after the femoral fracture in each group. Both in Piezo1*^f/f^* and in Piezo1*^Col2a1^* mice, compared with the blank hydrogels group, the C-LNP^@Fstl1^ Hydrogel group exhibited increased bone callus with trabecular coverage at the fracture ends (Figure 9B), along with an increase in BV/TV, Tb.N, and Tb.Th, and the Tb.Sp was decreased (Figure 9C-F). These results suggest that the inhibition of Fstl1 can reduce inflammatory cytokine in callus tissue and then reverse the delay in fracture healing caused by Piezo1 deficiency. Taken together, this study has elucidated the molecular mechanism by which the Piezo1-Fstl1 signaling axis regulates endochondral ossification during fracture healing via the inflammatory response (Figure 9G).

## Discussion

Fracture healing represents a complex histobiological and biochemical process that, depending on injury location, mechanism, and fixation method, progresses through two pathways analogous to skeletal development: intramembranous ossification and endochondral ossification^27^, ^28^. Endochondral ossification serves as the dominant mechanism for long bone fracture repair, playing an indispensable role in mechanically unstable fractures^29^. Research demonstrates this process forms a transient cartilaginous callus, a staged repair strategy offering dual benefits^30^: it provides mechanical stabilization through its proteoglycan-rich cartilaginous matrix that buffers fracture ends against early mechanical stress^31^, ^32^, while also orchestrating cascade regulation via chondrocyte-secreted VEGF and MMP-13 which mediate vascular invasion and calcified matrix resorption to establish a microenvironment conducive to chondrocyte-to-osteoblast transdifferentiation and subsequent bone replacement^33^, ^34^. Studies indicate that BMP and Hedgehog signaling promote endochondral ossification in fibrodysplasia ossificans progressiva^35^. Consequently, targeting critical regulatory nodes (e.g., enhancing transdifferentiation) holds significant promise as a novel therapeutic strategy for refractory fractures.

Mechanosensitive ion channel Piezo1 plays a pivotal role in endochondral ossification during fracture healing^36^-^38^. Clinical evidence confirms that early controlled motion increases stress loading at fracture sites, thereby enhancing healing^39^, ^40^. In murine femoral fracture models, postoperative mechanical stimulation accelerates callus formation, an effect directly linked to upregulated Piezo1 expression in mice^41^. Discovered by Coste *et al.* through RNAi screening in Neuro2A cells, Piezo1 serves as a critical mechanosensor in higher vertebrates^42^. It widely expresses across cell types, converting extracellular mechanical forces into Ca²⁺ influx to trigger diverse biological responses, including bone formation/remodeling and blood pressure homeostasis^6^, ^43^. Recent studies confirm high Piezo1 expression in osteoblastic lineages, where it is essential for skeletal mechanotransduction^44^. Cyclic tensile strain upregulates Piezo1 levels in chondrocytes^45^. Following mechanical stimulation of murine articular chondrocytes, Piezo1 expression is markedly elevated on the cell membrane, and calcium imaging detects significantly enhanced Ca²⁺ influx^46^. Pharmacological activation of Piezo1 using specific agonist Yoda1 promotes chondrocyte osteogenic differentiation.

Inflammatory response induced increased ROS in chondrocytes, leading to mitochondrial oxidative stress that hindered the endochondral ossification process in fracture healing^47^. Mitochondria are essential organelles involved in complex energy metabolism processes and play crucial roles in cell survival and execution of cellular functions. Stimulation of mitochondrial OxPhos has been reported to enhance bone synthesis metabolism and promote fracture repair^48^, ^49^. Mitochondrial dysfunction triggers chondrocyte apoptosis, with TUNEL assays revealing significantly elevated apoptosis rates following IL-1β stimulation^50^. Inflammatory responses induce ROS accumulation, which suppresses mitophagy (e.g., upon LPS stimulation), leading to damaged mitochondrial accumulation and excessive ROS production^51^, ^52^. Furthermore, ROS overload causes mitochondrial membrane potential collapse and mPTP opening, promoting mitochondrial fission while inhibiting fusion^53^. This membrane permeability shift results in mitochondrial matrix swelling, functional failure (reduced ATP synthesis and dysregulated Ca²⁺ homeostasis), and aberrant expression of mitochondrial dynamics proteins. ROS additionally activates NF-κB signaling to upregulate matrix metalloproteinases, accelerating cartilage matrix degradation and compromising the microenvironment essential for subsequent ossification^54^. Collectively, the inflammation-ROS-mitochondrial damage cascade directly induces chondrocyte death and indirectly impedes endochondral ossification by disrupting cellular metabolism and matrix homeostasis. This study investigates the crosstalk network among Piezo1-Fstl1 signaling axis, chondrocyte inflammation, mitochondrial function, and osteogenic differentiation, elucidating its pathoregulatory mechanism in fracture healing.

We pioneer the discovery that the Piezo1-Fstl1 signaling pathway regulates fracture healing by modulating chondrocyte inflammation. ScRNA-seq of callus tissues from Piezo1*^Col2a1^* and Piezo1*^f/f^* mice at postoperative day 14 revealed impaired endochondral ossification and significantly elevated Fstl1 expression in chondrocytes following Piezo1 deficiency. Fstl1, a novel pro-inflammatory mediator, orchestrates diverse biological processes (development, proliferation, differentiation)^55^. Under inflammatory conditions, it suppresses osteogenic differentiation of murine mesenchymal stem cells, though the specific matrix remains undefined^56^. In osteoarthritis, Fstl1 exacerbates chondrocyte apoptosis via the SAPK/JNK/Caspase-3 pathway and promotes cartilage matrix degradation through NF-κB activation, identifying it as a therapeutic target^57^. It has been reported to induce an inflammatory response in chondrocytes, microglia and preadipocytes, but no direct evidence of Fstl1's role in human bone injury was identified. Using shRNA plasmids to knock down Fstl1 in ATDC5 cells, we demonstrate that suppressing Fstl1 expression rescues Piezo1 deficiency-induced effects: reducing inflammatory cytokines, alleviating mitochondrial oxidative stress, restoring mitochondrial function, and promoting chondrocyte-to-osteoblast transdifferentiation.

Building upon LNPs—submicron delivery vectors self-assembled from phospholipids, cholesterol, and auxiliary lipids that enable efficient encapsulation and controlled delivery of nucleic acids or small molecules^58^—we encapsulated shFstl1 plasmids (LNP^@shFstl1^) for therapeutic intervention. Innovatively, surface functionalization with a CAP yielded third-generation targeting C-LNP^@shFstl1^, overcoming biological delivery barriers. Compared with LNP^@Fstl1^, the particle size of C-LNP^@Fstl1^ modified with targeted peptide was slightly larger (133.9 nm vs. 121.6 nm) and the degree of surface irregularity was slightly increased, but the structure of multilayer lipid particles with clear boundaries was still maintained. The HA-PBA/TA self-healing hydrogel is an intelligent biomaterial formed through synergistic crosslinking of HA-PBA and TA via dynamic boronate ester bonds and multiple hydrogen bonds^59^. Its self-healing capability originates from the dynamic reversible bonding of boronate esters, enabling rapid autonomous repair within seconds to minutes under physiological conditions, coupled with excellent biocompatibility and tissue adhesion^60^. Additionally, we observed that the hydrogel exhibits significant shear-thinning behavior due to force-dependent disassembly of internal crosslinks under increasing shear stress, ultimately achieving a liquid-like state and confirming its injectability. C-LNP^@Fstl1^ release and hydrogel degradation studies under simulated physiological conditions (37°C, PBS) demonstrated an initial burst release of approximately 25% of C-LNP^@Fstl1^ within the first day, followed by a gradual release rate that stabilized by day 10, while the hydrogel underwent 89.9% degradation within 14 days, indicating its capacity for temporally controlled drug release. By integrating cartilage targeting with non-viral lipid nanoparticles-composed of clinically approved MC3 lipid material-this study achieves a balance among safety, targeting efficiency, and gene silencing efficacy, offering an improved non-viral delivery strategy for fracture gene therapy.

This study has some limitations. First, although our results revealed that Piezo1 deletion inhibited fracture healing by upregulating Fstl1 expression, the nature of the intermolecular interactions between this mechanosensitive channel and Fstl1 remains unknown and requires further investigation. Second, the animal experiments conducted in this study utilized male mice. We have not yet included female mice in the current research, which represents a limitation of this study. In follow-up studies, we plan to employ female mice to investigate whether sex differences affect the experimental results. Finally, the therapeutic efficacy of our C-LNP^@Fstl1^ self-healing hydrogel has so far been validated only in murine models. Subsequent studies will extend these investigations to non-human primates to further evaluate the treatment outcomes of this intelligently responsive nanomaterial and advance its clinical translation.

In summary, this study confirms that the Piezo1-Fstl1 signaling pathway modulates chondrocyte inflammation and ROS accumulation, thereby inducing mitochondrial oxidative stress and functional impairment, ultimately affecting endochondral ossification. Furthermore, we developed an HA-PBA/TA self-healing hydrogel loaded with C-LNP^@Fstl1^ for targeted *in vivo* therapy, which markedly enhanced fracture healing in Piezo1*^Col2a1^* mice. Therefore, we propose that the Piezo1-Fstl1 signaling pathway holds the potential to be a novel therapeutic target for accelerating fracture healing.

## Supplementary Material

Supplementary figures and tables.

## Abbreviations

ROS: reactive oxygen species
MMP: mitochondrial membrane potential
mPTP: mitochondrial permeability transition pore
Fstl1: follistatin-like protein 1
OPA1: optic atrophy 1
MFN1: mitofusin 1
MFN2: mitofusin 2
DRP1: dynamin-related protein 1
COL1: collagen type I
OPN: osteopontin
RUNX2: runt-related transcription factor 2
OCN: osteocalcin
HA-PBA: hyaluronic acid-3-aminophenylboronic acid
TA: tannic acid
C-LNP^@Fstl1^: chondrocyte-targeting lipid nanoparticles incorporating Fstl1-shRNA
CCK-8: cell counting kit-8
BMP2: bone morphogenetic protein
ATP: adenosine triphosphate
ScRNA-seq: single-cell RNA sequencing
NCBI: National Center for Biotechnology Information
BV/TV: bone volume / total volume
Tb.N: trabecular number
Tb.Th: trabecular thickness
Tb.Sp: trabecular separation
HE: hematoxylin-eosin
SO/FG: Safranin O and Fast Green
IHC: immunohistochemistr
TEM: transmission electron microscope
SEM: scanning electron microscope
