# Supplementary material for: Piezo1-Fstl1 Axis in Fracture Healing: Modulation of the Chondrocyte Inflammation-ROS-Mitochondrial Damage Cascade and Application of Smart Delivery System
Source: Int J Biol Sci. 2026 Feb 18;22(6):2806–26. doi: 10.7150/ijbs.125814 (PMC13050443; doi:10.7150/ijbs.125814)
Supplement: Supplementary file 1 — Supplementary figures and tables. [file ijbsv22p2806s1.pdf]

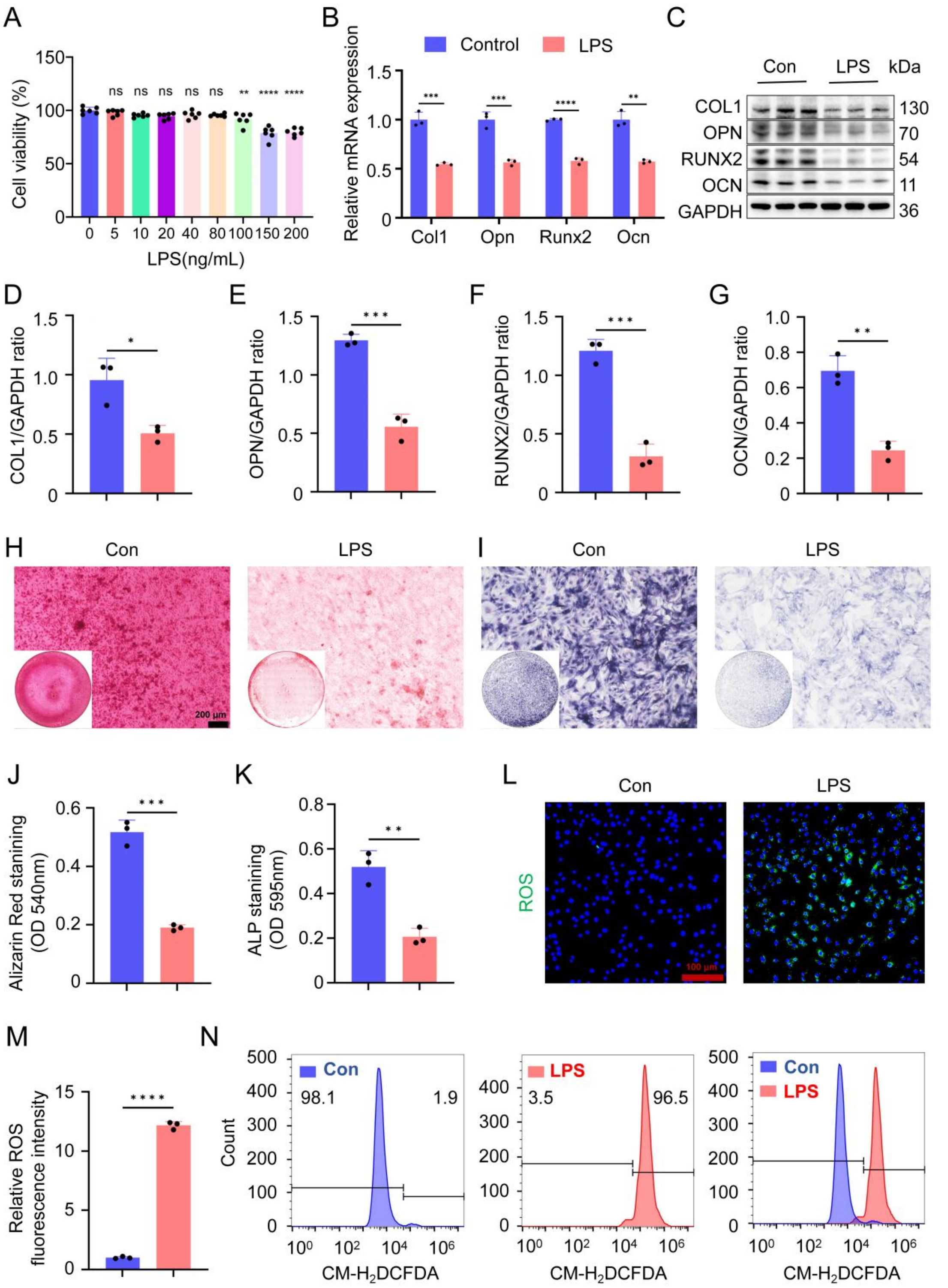

Figure S1. Inflammatory response induced chondrocyte ROS increase and hinder endochondral ossification *in vitro*. (A) Cytotoxicity experiments were performed to detect the effects of different concentrations of LPS on chondrocytes to determine the optimal concentration

of LPS (Data are represented as means ± SD, n = 6 per group, ANOVA was performed to compare data between groups, **P < 0.01, P < 0.0001, and ns > 0.05). **(B)** qPCR was used to detect changes in the expression of osteogenic markers Col1, Opn, Runx2, and Ocn in each group after osteogenic induction culture (Data are represented as means ± SD, n = 3 per group, t-test was performed, **P < 0.01, ***P < 0.001, and ****P < 0.0001). **(C-G)** WB was used to detect changes in the expression of osteogenic markers COL1, OPN, RUNX2, and OCN in each group after osteogenic induction culture and statistical analysis (Data are represented as means ± SD, n = 3 per group, t-test was performed, *P < 0.05, **P < 0.01, and ***P < 0.001). **(H, I)** Alizarin red and ALP staining were used to detect the expression of calcium nodules after 21 days of induction culture and the ALP levels after 7 days of osteogenic induction culture, scale = 200 μm. **(J, K)** Statistical analysis of Alizarin red and ALP staining were performed (Data are represented as means ± SD, n = 3 per group, t-test was performed, **P < 0.01, and ***P < 0.001). **(L, M)** ROS staining was used to detect the fluorescence intensity of CM-H2DCFDA in each group, and statistical analysis was performed, scale = 100 μm (Data are represented as means ± SD, n = 3 per group, t-test was performed, ****P < 0.0001). **(N)** Representative flow cytometry image of mean fluorescence intensity of ROS in each group.

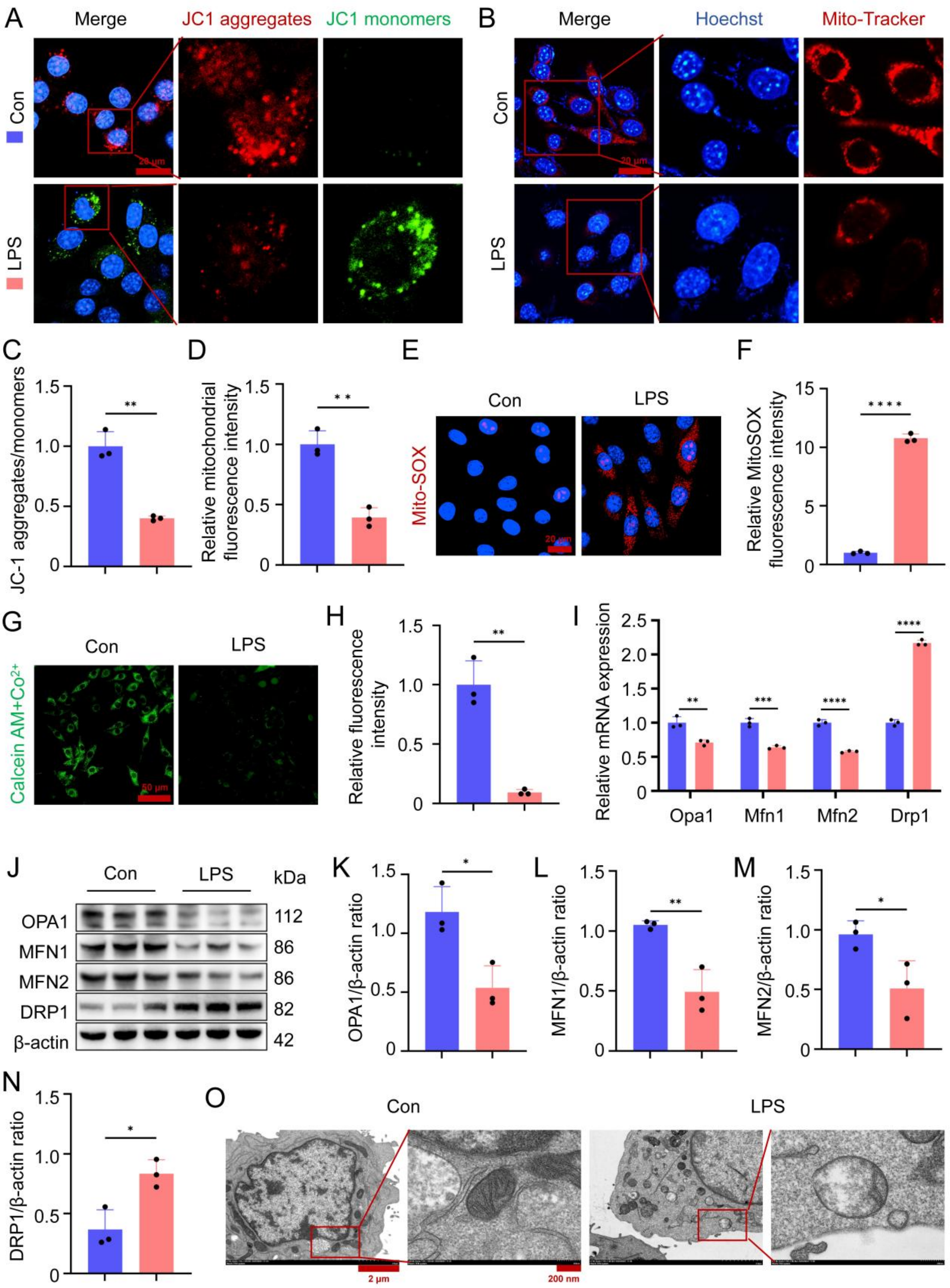

Figure S2. Inflammatory response induced mitochondrial oxidative stress, mPTP opening and dysfunction. (A) JC-1 staining was used to detect changes in mitochondrial membrane potential in each group, scale = 20 μm. Blue fluorescence indicates the nuclei of live cells, red fluorescence indicates that JC-1 exists in the form of polymers (J-aggregates) in mitochondria, and green

fluorescence indicates that JC-1 exists in the form of monomers in the mitochondria. **(B)** Mito-Tracker Red staining was used to detect fluorescence changes in biologically active mitochondria in each group, scale = 20 μm. Red fluorescence indicates mitochondria with biological activity, and blue fluorescence indicates the nuclei of live cells. **(C, D)** Statistical analysis of fluorescence intensity of JC-1 and Mito-Tracker Red staining (Data are represented as means ± SD, n = 3 per group, t-test was performed, ** $P < 0.01$). **(E, F)** MitoSOX Red was used to detect changes in the expression of superoxide in the mitochondria of each group and statistical analysis, scale = 20 μm (Data are represented as means ± SD, n = 3 per group, t-test was performed, **** $P < 0.0001$). Blue fluorescence indicates the nuclei of live cells; red fluorescence represents the oxidation product formed by the reaction with MitoSOX Red. **(G, H)** mPTP Assay Kit was used to detect changes in the expression of Calcein green fluorescence in the mitochondria of each group and statistical analysis, scale = 50 μm (Data are represented as means ± SD, n = 3 per group, t-test was performed, ** $P < 0.01$). **(I)** qPCR was used to detect the expression of mitochondrial dynamics-related genes (Opa1, Mfn1, Mfn2, and Drp1) in each group (Data are represented as means ± SD, n = 3 per group, t-test was performed, ** $P < 0.01$, *** $P < 0.001$, and **** $P < 0.0001$). **(J-N)** WB was used to detect changes in the expression of mitochondrial dynamics-related markers (OPA1, MFN1, MFN2, and DRP1) in each group, and statistical analysis (Data are represented as means ± SD, n = 3 per group, t-test was performed, * $P < 0.05$, and ** $P < 0.01$). **(O)** TEM was used to observe the mitochondrial microstructure and ultrafine details of the cells in each group, scale = 2 μm (left); 200 nm (right).

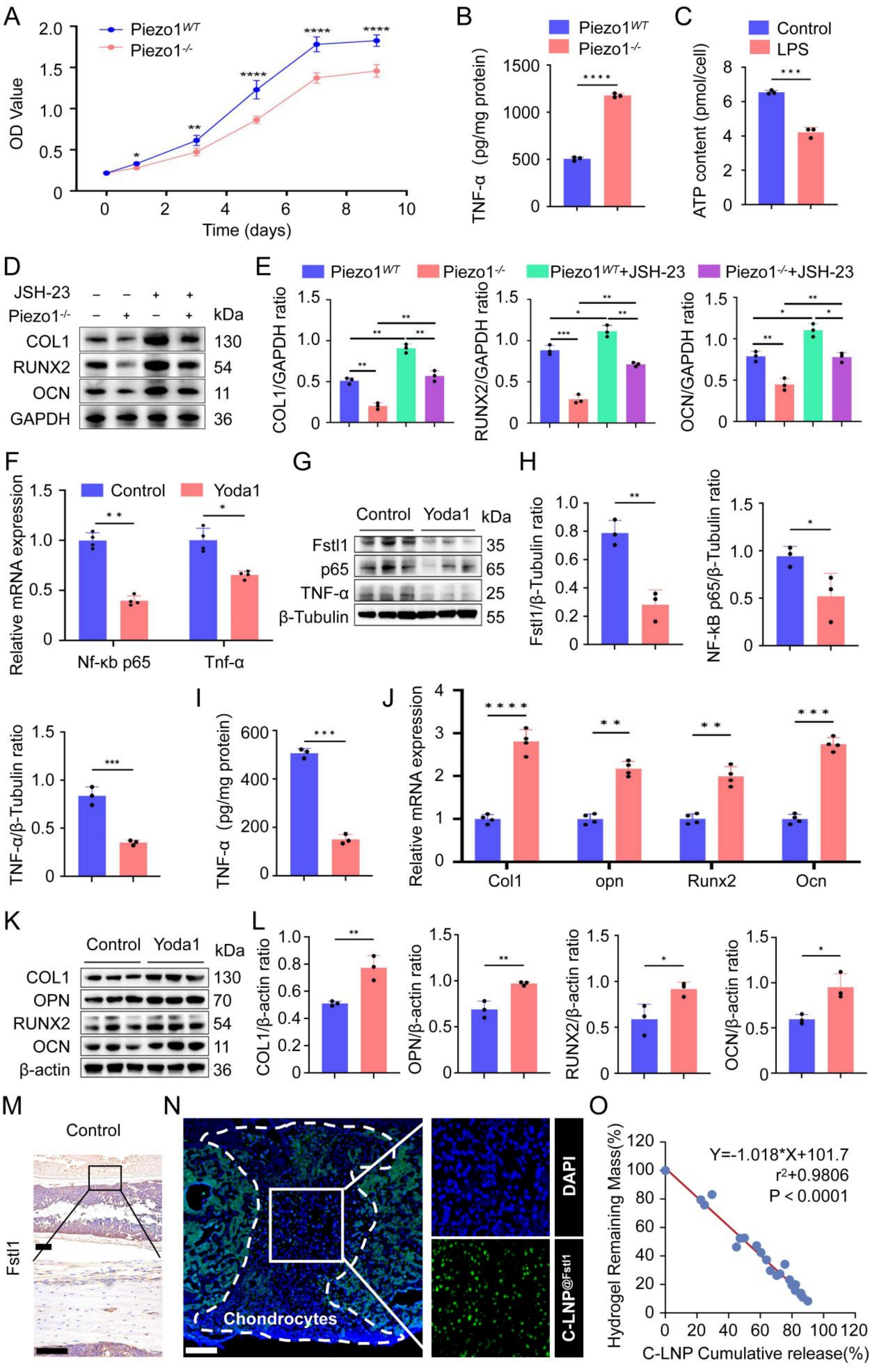

Figure S3. Supplementary Material. (A) Proliferation of Piezo1^{WT} and Piezo1$^{-/-}$ ATDC5 cells was

quantitatively evaluated using the CCK-8 assay. **(B)** TNF-α levels in the culture medium of Piezo1^{WT} and Piezo1$^{-/-}$ ATDC5 cells were assessed using ELISA (Data are represented as means ± SD, n = 3 per group, t-test was performed, ****P < 0.0001). **(C)** An ATP assay kit was used to detect ATP levels in each group (Data are represented as means ± SD, n = 3 per group, t-test was performed, ***P < 0.001). **(D, E)** WB was used to detect changes in the expression of osteogenic markers COL1, RUNX2, and OCN in each group after osteogenic induction culture and statistical analysis (Data are represented as means ± SD, n = 3 per group, ANOVA was performed to compare data between groups, *P < 0.05, **P < 0.01, and ***P < 0.001). **(F)** qPCR was used to detect changes in the expression of inflammatory related factors Nf-κb p65 and Tnf-α in each group (Data are represented as means ± SD, n = 4 per group, t-test was performed, *P < 0.05, and **P < 0.01). **(G, H)** WB was used to detect changes in the expression of Fstl1 and inflammatory related proteins NF-κB p65, and TNF-α in each group after osteogenic induction culture and statistical analysis (Data are represented as means ± SD, n = 3 per group, t-test was performed, *P < 0.05, **P < 0.01, and ***P < 0.001). **(I)** TNF-α levels in the culture medium of control and Yoda1 ATDC5 cells were assessed using ELISA (Data are represented as means ± SD, n = 3 per group, t-test was performed, ***P < 0.001). **(J)** qPCR was used to detect changes in the expression of osteogenic markers Col1, Opn, Runx2, and Ocn in each group after osteogenic induction culture (Data are represented as means ± SD, n = 4 per group, t-test was performed, **P < 0.01, ***P < 0.001, and ****P < 0.0001). **(K, L)** WB was used to detect changes in the expression of osteogenic markers COL1, OPN, RUNX2, and OCN in each group after osteogenic induction culture and statistical analysis (Data are represented as means ± SD, n = 3 per group, t-test was performed, *P < 0.05, and **P < 0.01). **(M)** IHC staining was used to detect the baseline level of Fstl1 in non-fractured tissues, scale = 500

μm (up) and 200 μm (down). **(N)** Tissue fluorescence staining to validate GFP-C-LNP$^{@Fstl1}$ targeted accumulation in chondrocytes within the callus, scale = 200 μm. **(O)** The correlation analysis between the degradation kinetics of the HA-PBA/TA hydrogel and the release behavior of C-LNP$^{@Fstl1}$.

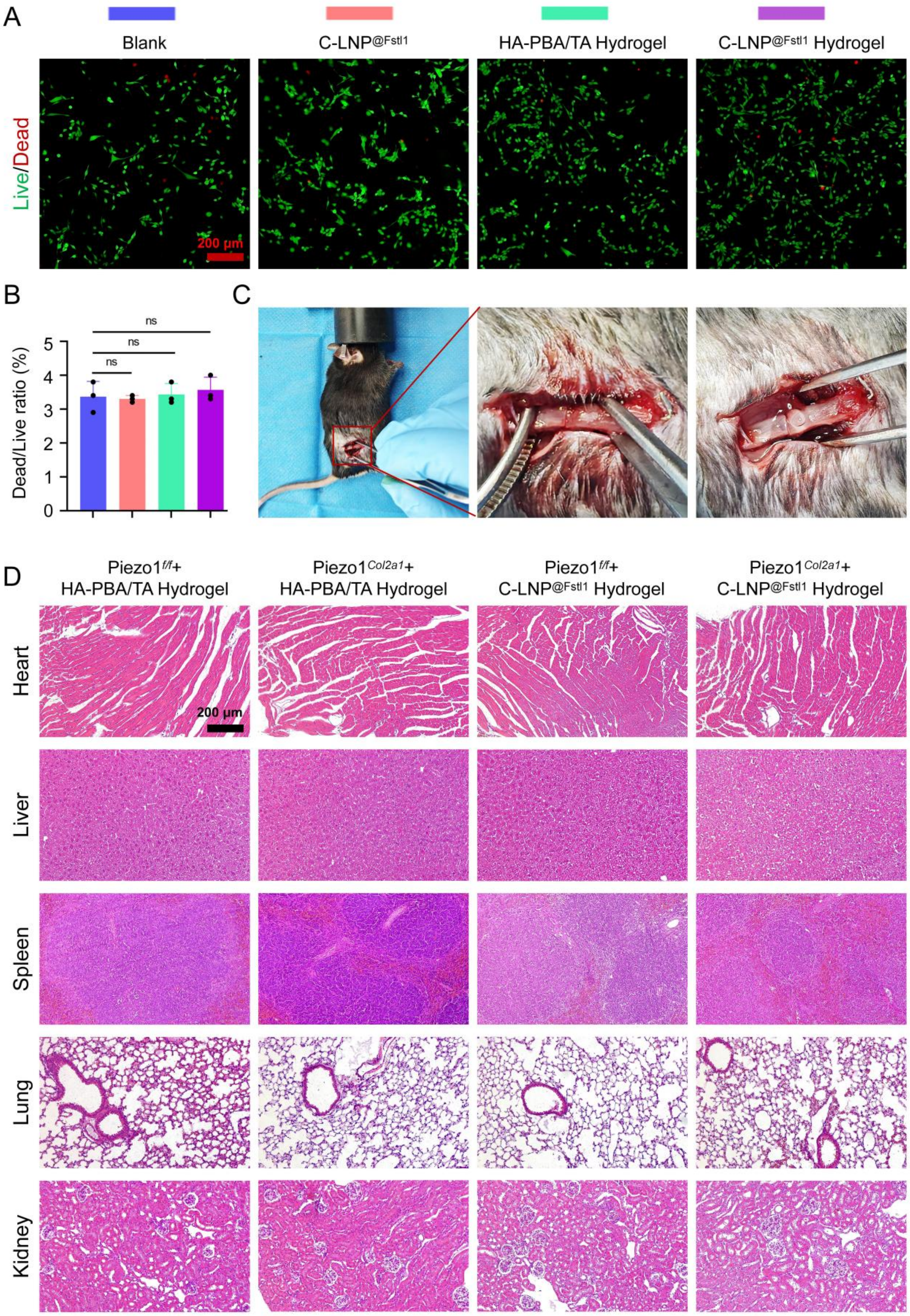

Figure S4. Safety assessment of C-LNP$^{@Fstl1}$ and HA-PBA/TA hydrogel in *vitro* and in *vivo*. (A, B) Effect of live/dead cell staining detection C-LNP$^{@Fstl1}$, HA-PBA/TA hydrogel and C-LNP$^{@Fstl1}$ hydrogel on cytotoxicity, scale = 200 μm (Data are represented as means ± SD, n = 3 per group,

ANOVA was performed to compare data between groups, ns > 0.05). **(C)** The hydrogel via local injection at murine femoral fracture sites, n = 5 mice per group. **(D)** HE staining was used to detect the histological differences or pathological alterations of major visceral organs (heart, liver, spleen, lung, and kidney) in each group of mice, scale = 200 μm, n = 5 mice per group.

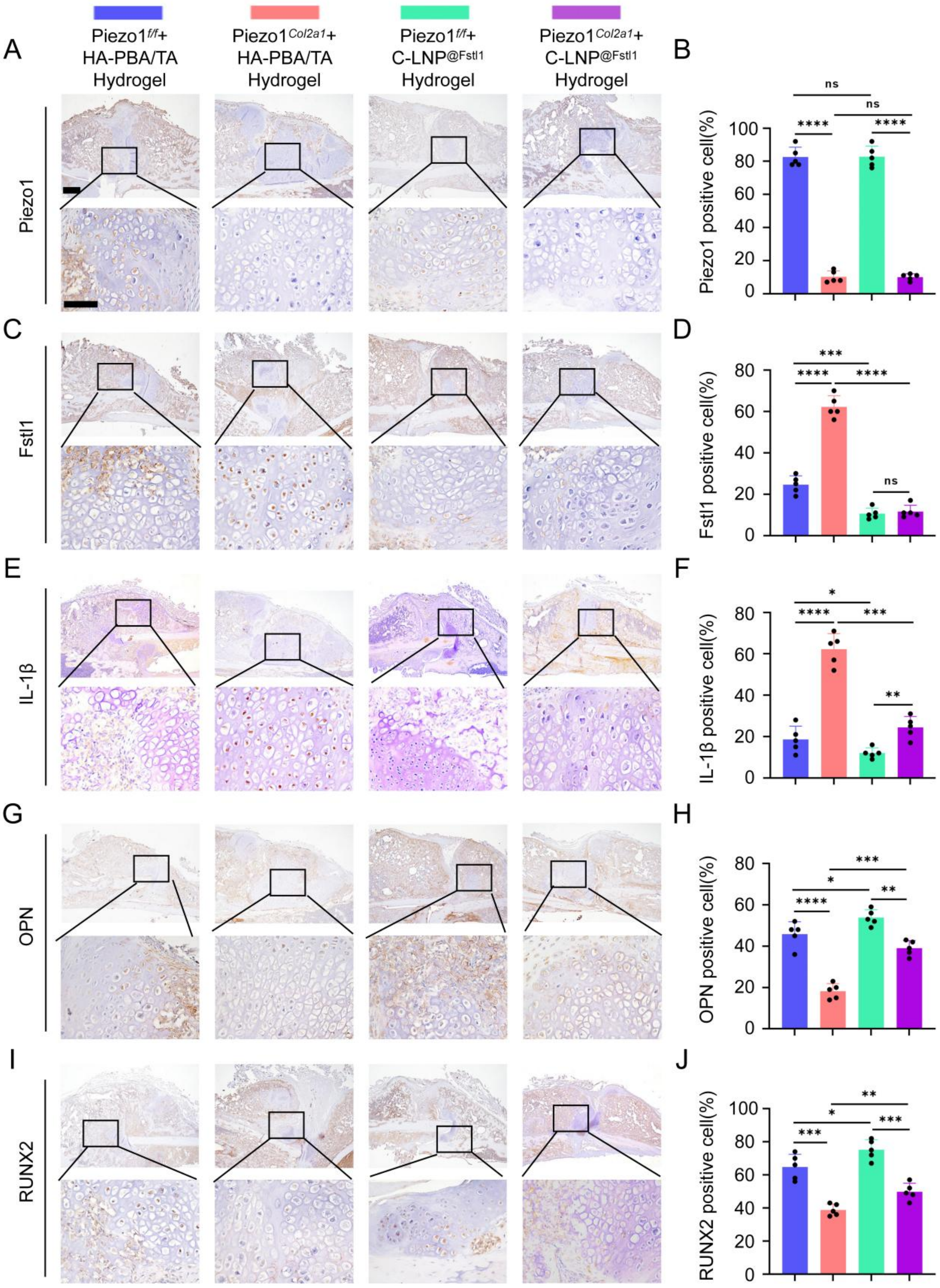

Figure S5. The HA-PBA/TA self-healing hydrogel loaded with C-LNP$^{@Fstl1}$ reduced inflammatory cytokine and increased osteogenic factors in callus tissue. (A, B) IHC staining was used to detect the expression of Piezo1 in hypertrophic chondrocytes of callus in each group, with statistical analysis, scale = 500 μm (up) and 200 μm (down); (Data are represented as means ±

SD, n = 5 mice per group, ANOVA was performed to compare data between groups, ****$P < 0.0001$, and ns > 0.05). **(C, D)** IHC staining was used to detect the expression of Fstl1 in hypertrophic chondrocytes of callus in each group, with statistical analysis; (Data are represented as means ± SD, n = 5 mice per group, ANOVA was performed to compare data between groups, ***$P < 0.001$, ****$P < 0.0001$, and ns > 0.05). **(E, F)** IHC staining was used to detect the expression of inflammatory related proteins IL-1β in hypertrophic chondrocytes of callus in each group, with statistical analysis; (Data are represented as means ± SD, n = 5 mice per group, ANOVA was performed to compare data between groups, *$P < 0.05$, **$P < 0.01$, ***$P < 0.001$, and ****$P < 0.0001$). **(G-J)** IHC staining was used to detect the expression of osteogenic markers OPN, and RUNX2 in hypertrophic chondrocytes of callus in each group, with statistical analysis; (Data are represented as means ± SD, n = 5 mice per group, ANOVA was performed to compare data between groups, *$P < 0.05$, **$P < 0.01$, ***$P < 0.001$, ****$P < 0.0001$).

Table S1. Genotyping Conditions and PCR Primers

PrimerName	Primer sequence (5'-3')
Piezo1$^{f/f}$-F	CCAGTGATTCCTCATGGAATGTGG
Piezo1$^{f/f}$-R	CTTAAGCCCATCTCACAGCTGAAGG
Col2a1-CreERT2-F	GGCTCTACTTCATCGCATTCCTTG
Col2a1-CreERT2-R	CGCAAACAAGTCTCACAAAGGAG

Table S2. Fstl1 interferes with the gene sequence of the plasmid

shRNA	Primer sequences
Fstl1-RNAi-1(PSC112876-1)	GCAGAATGAAACAGCCATCAA
Fstl1-RNAi-2(PSC112877-1)	GAAGGTGAACACCAAAGAGAT
Fstl1-RNAi-3(PSC112878-1)	CTCTGCATTGAGCAATGCAAA

Table S3. Sequences of primers used for RT-PCR

Gene	Forward primer (5′-3′)	Reverse primers (5′-3′)
Piezo1	GAATGTGATTGGGCAGCGTATGAAC	GAACAGCGTGAGGAACAGACAGTAG
Fstl1	TTATGATGGGCACTGCAAAGAA	ACTGCCTTTAGAGAACCAGCC
Col1a1	AGGCGAACAAGGTGACAGAGG	GGAGAACCAGGAGAACCAGGAG
Opn(Spp1)	TTCAATGGGCAGTTTTGAGC	ACTTACAAAACCGCCAAGC
Runx2	CCGCACGACAACCGCACCAT	CGCTCCGGCCCACAAATCTC
Ocn	AAGCAGGAGGGCAATAAGGT	TTTGTAGGCGGTCTTCAAGC
Nf-κb	CGAGTCTCCATGCAGCTACG	TTTCGGGTAGGCACAGCAATA
Tnf-α	TGGCCTCCCTCTCATCAGTT	TTGAGATCCATGCCGTTGGC
Il-1β	TCCAGGATGAGGACATGAGCAC	GAACGTCACACACCAGCAGGTTA
Mfn1	GTGGGCTGGAAACTAATCTCTGTC	AACTGCTGCTTAAACGCTCTCTC
Mfn2	GTGGTCGGAGGAGTGGTGTG	GTGCCAGGTCAGTCGCTCATAG
Opa1	ATGACAGAACCCAAAGGAAAGGAAC	CCACTTGTGCCGCTTGATACTC
Drp1	TGGCAACATCAGAAGCACTCAAG	TGGCATCAGTACCCGCATCC
Gapdh	GGGGAGCCAAAAGGGTCATCATCT	GAGGGGCCATCCACAGTCTTCT
